# Analysis of the BarA/UvrY Two-Component System in *Shewanella oneidensis* MR-1

**DOI:** 10.1371/journal.pone.0023440

**Published:** 2011-09-12

**Authors:** Lucas Binnenkade, Jürgen Lassak, Kai M. Thormann

**Affiliations:** Department of Ecophysiology, Max-Planck-Institut für Terrestrische Mikrobiologie, Marburg, Germany; Universität Münster, Germany

## Abstract

The BarA/UvrY two-component system is well conserved in species of the γ-proteobacteria and regulates numerous processes predominantly by controlling the expression of a subset of noncoding small RNAs. In this study, we identified and characterized the BarA/UvrY two-component system in the gammaproteobacterium *Shewanella oneidensis* MR-1. Functional interaction of sensor kinase BarA and the cognate response regulator UvrY was indicated by *in vitro* phosphotransfer studies. The expression of two predicted small regulatory RNAs (sRNAs), CsrB1 and CsrB2, was dependent on UvrY. Transcriptomic analysis by microarrays revealed that UvrY is a global regulator and directly or indirectly affects transcript levels of more than 200 genes in *S. oneidensis*. Among these are genes encoding key enzymes of central carbon metabolism such as *ackA*, *aceAB*, and *pflAB*. As predicted of a signal transduction pathway that controls aspects of central metabolism, mutants lacking UvrY reach a significantly higher OD than the wild type during aerobic growth on N-acetylglucosamine (NAG) while under anaerobic conditions the mutant grew more slowly. A shorter lag phase occurred with lactate as carbon source. In contrast, significant growth phenotypes were absent in complex medium. Based on these studies we hypothesize that, in *S. oneidensis* MR-1, the global BarA/UvrY/Csr regulatory pathway is involved in central carbon metabolism processes.

## Introduction

The genus *Shewanella* belongs to the γ-proteobacteria and is characterized by a remarkable respiratory diversity. An enormous range of alternative terminal electron acceptors can be used in the absence of oxygen, including nitrogen- and sulfur-containing compounds and soluble or insoluble metal oxides such as Fe(III) and Mn(III/IV) [1], [2]. This respiratory flexibility is thought to enable species of the genus to thrive in redox-stratified environments [3]. *Shewanella* species have been isolated from a wide range of different habitats, and members of this genus have been implicated in diverse roles such as causative agents of food spoilage, opportunistic pathogens or, on the other hand, as potential agents in bioremediation or microbial fuel cells [4], [5], [6], [7]. However, to fully capitalize on the biotechnological potential of *Shewanella*, it is necessary to understand how members of this genus respond to changing environmental conditions. Accordingly, a number of studies have addressed regulatory systems underlying metabolic and respiratory processes in this genus, in particular that of the species *S. oneidensis* MR-1.

Key regulatory systems that have been characterized in other species of the γ-proteobacteria, such as the enteric bacteria, can be readily identified in *Shewanella*. However, despite a high degree of conservation at the amino acid level in the regulatory components, the output was demonstrated to be remarkably different from systems previously described in other bacteria. In *Escherichia coli*, FNR and the Arc two-component system are major regulators in the adaptation of the metabolism to changing oxygen levels [8], [9], [10]. In *S. oneidensis* MR-1, the corresponding orthologous systems, EtrA and ArcS/HptA/ArcA, only have a minor role in that process [11], [12], [13], and the corresponding regulons differ significantly between *S. oneidensis* MR-1 and *E. coli* [12], [13], [14], [15]. Interestingly, the cyclic AMP receptor protein (CRP) is thought to mainly regulate the metabolic adaptation during the shift to anaerobic conditions in *Shewanella* in response to cAMP levels [16], [17]. In *E. coli*, the cAMP/CRP regulation cascade has become the paradigm system for catabolite repression in Gram-negative bacteria [18].

Another global regulating unit that is well conserved among γ-proteobacteria is the BarA/UvrY (*E. coli*) or GacS/GacA (*Pseudomonas*) two-component system [19]. BarA/GacS is a membrane-located sensor kinase that autophosphorylates upon perception of a signal and transfers a phosphoryl group to the cognate response regulator UvrY/GacA. Intermediates of the Krebs cycle or acetate and formate have been demonstrated to stimulate the regulating cascade in *P. fluorescens* and *E. coli*, respectively. However, it remains to be shown if these compounds are the direct signal [20], [21]. Phosphorylation of the response regulator is thought to result in dimerization and binding to the corresponding target promoter region [22], [23], [24]. Notably, BarA/UvrY (GacS/GacA) has been demonstrated to act predominantly, or even exclusively [25], via control of expression of several small RNA molecules (Csr or Rsm). Upon expression, these sRNAs effectively bind and thereby antagonize the effect of translational regulator proteins (CsrA or RsmA/E) that often block the Shine-Dalgarno sequence of corresponding target genes. In *E. coli*, another protein, CsrD, is involved in the regulatory cascade by specifically targeting the regulatory RNAs for degradation by RNase E [26]. The output of the BarA/UvrY (GacS/GacA) and homologous regulatory systems is highly diverse. It controls the production of extracellular factors, such as exoenzymes or toxins, quorum sensing, motility, and diverse metabolic functions. Thus, for many bacterial species, BarA/UvrY (GacS/GacA) is critical for regulation and coordination of pathogenicity and group behaviors [19].

Genome annotation and bioinformatic analyses in *S. oneidensis* MR-1 indicate the presence of putative orthologs to BarA, UvrY, CsrA and the Csr sRNAs [5], [27], [28]. This strongly suggests the existence of a BarA/UvrY/Csr pathway in *Shewanella*. In this study, we used phosphotransfer studies to demonstrate that the *S. oneidensis* orthologs to BarA and UvrY constitute a two-component system and transcriptomic studies to identify the UvrY regulon. We further provide evidence that at least two putative sRNAs are regulated by UvrY and BarA, and finally that the pathway regulates carbon metabolism in *Shewanella*.

## Results

### Identification of a BarA/UvrY two-component system in *S. oneidensis* MR-1

**Figure 1:**
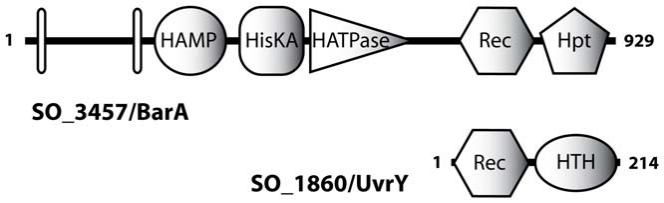
Domain organization of *S. oneidensis* MR-1 BarA (SO_3457; upper panel) and UvrY (SO_1860, lower panel). White vertical bars mark the positions of transmembrane domains. HAMP, HAMP signal transduction domain; HisKa, histidine kinase dimerization domain; HATPase, histidine kinase ATPase domain; Rec, receiver domain; Hpt, histidine-containing phosphotransfer domain; HTH, helix-turn-helix DNA-binding motif.

To identify potential orthologs to the *E. coli* BarA/UvrY or the *Pseudomonas* GacS/GacA systems in *S. oneidensis* MR-1, we performed a bioinformatic analysis on the genetic data available. Based on homology, SO_3457 and SO_1860 emerged as the most likely candidates. SO_3457, annotated as hybrid histidine kinase, is 2790 bp in length and encodes a protein of 929 amino acids with a predicted molecular mass of 103 kDa. At the amino acid level, SO_3457 shares 44% identity and 64% similarity to BarA of *E. coli*. SO_3457 is predicted to have two N-terminal transmembrane domains followed by a cytoplasmic HAMP domain, a histidine kinase A domain, an ATPase domain, a receiver domain, and a C-terminal histidine phosphotransfer domain (Fig. 1). This domain architecture equals that of BarA/GacS sensor kinases identified in other species. SO_3457 is likely transcribed in an operon with the downstream gene SO_3458, predicted to encode a conserved hypothetical protein of 199 amino acids.

SO_1860 is 645 bp in length, encoding a protein of 214 amino acids annotated as a response regulator with an N-terminal receiver and a C-terminal helix-turn-helix DNA-binding domain. At the amino acid level, SO_1860 shares 70% identity and 82% similarity to UvrY of *E. coli*. SO_1860 is likely to be the first gene in an operon with the downstream genes SO_1861 and SO_1862, predicted to encode a subunit of an exinuclease and a phosphatidylglycerophosphate synthetase, respectively. This gene order is also present for *uvrY* in *E. coli* and for *gacA* in *Pseudomonas* species. Notably, orthologs to both sensor kinase SO_3457 and response regulator SO_1860 are present in all *Shewanella* species sequenced so far (Table S1).

**Figure 2:**
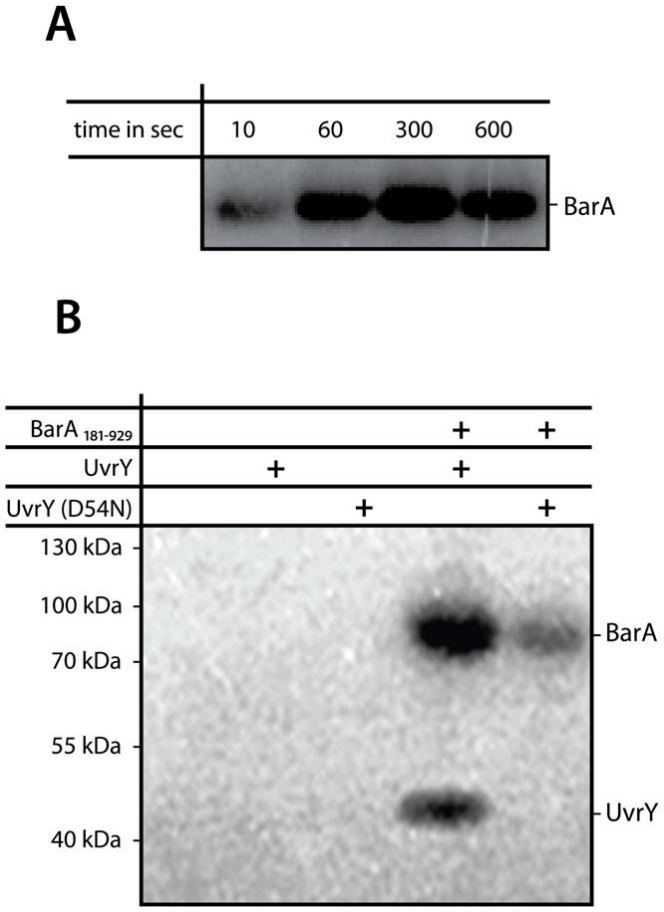
*In vitro* interaction of BarA and UvrY. A) Autoradiographic analysis of BarA autophosphorylation. B) Autoradiographic analysis of phosphotransfer between BarA(181–929) and UvrY. Purified BarA181–929, UvrY, or UvrY(D54N) were added to the reactions as indicated and incubated for 60 s.

While UvrY is highly conserved between *S. oneidensis* MR-1 and *E. coli* and resides in similar genetic context, the putative BarA is less well conserved. Also the genetic context in both species is different. We therefore determined whether the two components identified in *S. oneidensis* MR-1 in fact constitute a cognate sensor histidine kinase/response regulator system. To this end, we conducted *in vitro* phosphotransfer studies on purified proteins to further determine whether functional interactions occur between BarA and UvrY [23]. The cytoplasmic region of BarA (aa 181–929) was purified by using a recombinant N-terminal His tag fusion and UvrY by using a recombinant N-terminal GST fusion. We tested the activity of the purified sensor kinase by incubation with [γ-^32^P]ATP and subsequent SDS-PAGE separation (Fig. 2; Fig. S1). BarA was readily phosphorylated, indicating autophosphorylation activity of the kinase region. In contrast, GST-UvrY was not phosphorylated upon incubation with [γ-^32^P]ATP. We also tested whether GST-UvrY can be phosphorylated using [γ-^32^P]-acetyl phosphate, however no phosphorylation occurred (data not shown). Incubation of BarA and GST-UvrY together in the presence of [γ-^32^P]ATP resulted in significant phosphotransfer to GST-UvrY. No phosphotransfer occurred when a GST-UvrY variant was used in which the predicted site of phosphorylation was substituted [GST-UvrY(D54N)], indicating specific phosphorylation at the predicted aspartic acid residue. Based on the results of these *in vitro* studies, we concluded that SO_3457/BarA acts as a sensor kinase for SO_1860/UvrY in *S. oneidensis* MR-1.

### Identification of sRNAs CsrB1 and CsrB2

**Figure 3:**
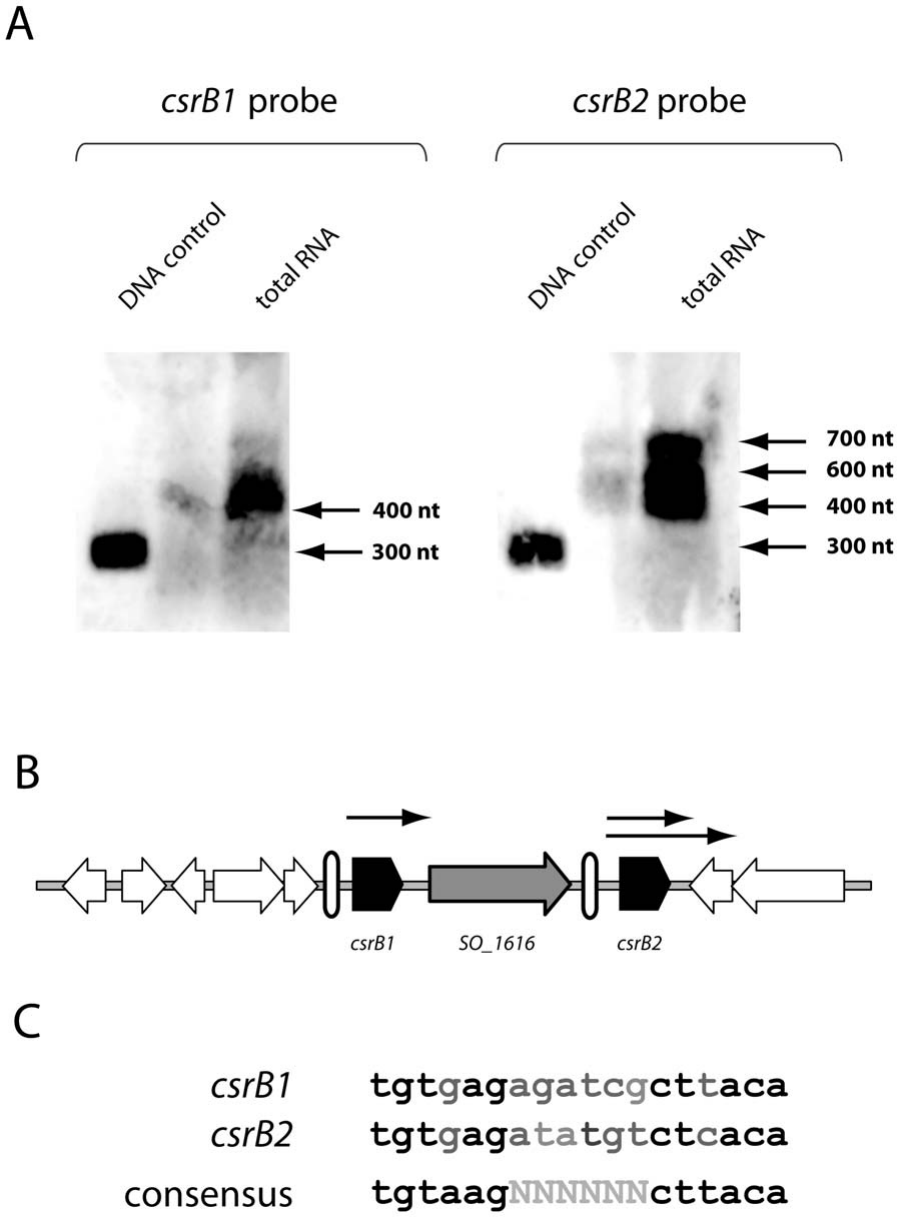
Analysis of the predicted sRNAs CsrB1 and CsrB2. A) Northern analysis of *csrB1* and *csrB2* transcripts. 15 µg total RNA isolated from *S. oneidensis* MR-1 LB cultures at late exponential growth phase were separated on a denaturing agarose gel. As a positive control, an unlabeled PCR product was used as s corresponding probe for *csrB1* or *csrB2* (DNA control). B) Genetic organization of the *S. oneidensis* MR-1 *csrB* locus. The predicted *csrB1* and *csrB2* genes are displayed in black, the surrounding genes in white. The *csrB1* and *csrB2* genes are separated by SO_1616, encoding a transposase. White vertical bars indicate the positions of predicted UvrY-binding boxes, black arrows putative transcripts. C) Putative UvrY-binding boxes upstream of *csrB1* and *csrB2*.

If SO_3457 and SO_1860 constitute the Shewanella BarA/UvrY pathway, then it should regulate the expression of Csr/RsmA sRNAs like other BarA/UvrY (or GacS/GacA) pathways [25], [29], [30], [31]. These sRNAs, in turn, antagonize the activity of the mRNA-binding regulator CsrA(RsmA). A CsrA ortholog, SO_3426, has already been annotated in *S. oneidensis* MR-1 due to its striking conservation levels (93% identity to *E. coli* CsrA). The presence of two *csrB* genes, *csrB1* (420 bp) and *csrB2* (421 bp), encoding putative corresponding regulatory sRNAs, has been predicted previously in the intergenic regions between SO_1615/SO_1616 and SO_1616/SO_1617, separated by SO_1616 encoding a transposase (Fig. 3) [28]. The prediction was based on numerous CsrA-binding AGGA/ARGGA motifs mainly occurring in single-stranded regions of the sRNA's secondary structure (Fig. S2). Furthermore, a putative UvrY/GacA-binding box (TGTAAGN_6_CTTACA) [32], [33], [34] was identified upstream of the putative *csrB1* and *csrB2* genes [28].

To determine if the two hypothetical sRNA-encoding genes *csrB1* and *csrB2* are transcribed and to determine the corresponding transcript length, we performed Northern blot analysis on total RNA using probes complementary to the corresponding gene. Total RNA was prepared from cells at late exponential phase grown in complex media. With either probe, distinct signals were observed (Fig. 3), strongly indicating expression of both predicted sRNAs. The major transcript of *csrB1* had a size of approximately 450 bp, nicely corresponding to the predicted size of 420 bp. In contrast, the transcript of *csrB2* occurs evenly in at least three different sizes at approximately 700, 600, and 450 bp. The transcript sizes exclude the occurrence of a large transcript including the transposase gene SO_1616 with a predicted size of 1203 bp and indicate that both sRNAs are transcribed separately. To determine whether the rather large transcript of *csrB2* is initiated from the transposase gene, we performed RT-PCR using primers bracketing the gap between SO_1616 and *csrB2*. No product was obtained (data not shown), indicating that no transcription of *csrB2* occurs from SO_1616.

### Putative CsrB1 and CsrB2 are regulated by BarA/UvrY

**Figure 4:**
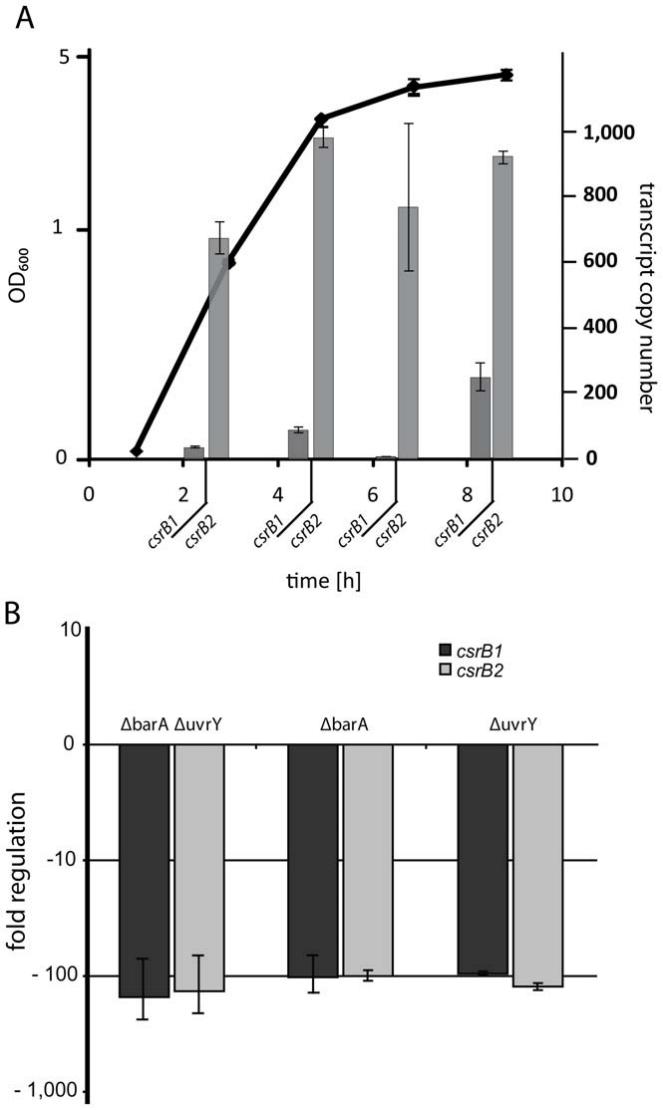
Analysis of *csrB1* and *csrB2* expression. A) Growth phase-dependent *csrB* expression in complex medium. Total RNA was prepared from cells at the indicated time points and used to calculate the transcript copy number by q-RT-PCR. The error bars display the standard deviation. B) Decrease in expression of *csrB1* and *csrB2* in *uvrY* and *barA* mutants. Total RNA was prepared from cells of the appropriate mutant strain at late exponential phase, and the mRNA levels of *csrB1* and *csrB2* were quantified by q-RT-PCR. The error bars display the standard deviation.

To determine if *csrB1* is transcribed at lower levels than *csrB2*, as suggested by Northern analysis, we performed q-RT-PCR using total RNA that was prepared from cells at different growth phases during aerobic growth in complex medium (Fig. 4). In wild-type cells, transcript levels of *csrB1* were significantly lower than those of *csrB2* (by a factor of 3–10), as was already indicated by the Northern analysis. The expression pattern of *csrB2* displayed only minor changes throughout growth while the *csrB1* transcription level increased during late exponential/early stationary phase. To determine whether *csrB1* and *csrB2* are regulated by the potential BarA/UvrY two-component system, we constructed in-frame deletions in *uvrY* (*ΔuvrY*), *barA* (*ΔbarA*), and in both (*ΔbarAΔuvrY*). Re-integration of the deleted gene section resulted in the wild-type phenotype, indicating that the observed mutant phenotypes were due to the deletion of the targeted genes (data not shown). The mutant and wild-type strains grew equally well in complex LB medium. In all growth phases examined, the expression levels of both *csrB1* and *csrB2* were drastically decreased by a factor of >100 to low levels in all mutants strains compared to those of the wild type (Fig. 4B). However, differences in *csrB1* levels depending on the growth phase still occurred (data not shown). This regulation pattern strongly indicates that *csrB1* and *csrB2* are controlled by both BarA and UvrY. Thus, taken together with the phosphotransfer studies, we concluded that BarA is the cognate sensor kinase for UvrY.

A third putative sRNA of *S. oneidensis* MR-1, CsrC, was predicted to be encoded between *mutM* and SO_4727. However, this region contains only four conserved AGGA/ARGGA motifs that are typical for sRNAs of the Csr/Rsm family [28], [35]. To determine whether transcription occurs from the predicted gene, q-RT-PCR was also performed using primers complementary to the corresponding gene region. Significant transcription of putative *csrC* was observed (data not shown). However, in contrast to *csrB1* and *csrB2*, the transcription level of the predicted *csrC* sRNA was not affected by the absence of BarA and/or UvrY (data not shown) which was inconsistent with a role in a BarA/UvrY regulatory system. Based on these results we concluded that at least two predicted sRNAs, CsrB1 and CsrB2, are likely components of a BarA/UvrY/Csr regulatory cascade in *S. oneidensis* MR-1.

### The regulon of UvrY in *S. oneidensis* MR-1

BarA/UvrY and orthologous systems have been demonstrated to represent global regulatory systems in γ-proteobacteria [19]. To elucidate the potential role of this system in *Shewanella*, we determined the impact of a *uvrY* deletion on the transcriptome of *S. oneidensis* MR-1 by microarray analysis. Total RNA was prepared from cells of the wild type and the *ΔuvrY* strain grown aerobically in LB medium to early stationary phase (OD_600_ of 4.0) and used for transcriptomic analysis by microarrays. The expression data obtained from microarray analysis was subsequently confirmed by q-RT-PCR on 5 genes displaying different levels of regulation (r^2^ = 0.949) (Fig. S3). According to the statistical analysis (*P*<0.05), 208 genes had significantly different transcriptional levels (log_2_≥1). Among these 208 genes, 80 genes were significantly up-regulated (listed in Table S2) and 128 genes were significantly down-regulated (listed in Table S3). About half of these regulated genes (103) encode proteins of unknown function and are particularly overrepresented among those upregulated in *ΔuvrY*. Other major functional groups of differently regulated genes encode proteins that are predicted to be involved in amino acid and carbohydrate transport and metabolism as well as energy production and conversion. Among the latter is, for example, AckA which is required for substrate level phosphorylation under anaerobic conditions in *S. oneidensis* MR-1 [36]. Other key enzymes of central carbon metabolism positively regulated by UvrY are AceA/AceB, instrumental for the glyoxalate cycle, and PflA and PflB, involved in the conversion of pyruvate to acetyl-CoA and formate. A large gene cluster encoding numerous glycosyl transferases (SO_4193 – SO_3171) is downregulated in the *ΔuvrY* mutant, indicating positive control of this putative operon by BarA/UvrY. The proteins encoded by this cluster are thought to be involved in cell envelope synthesis or exopolysaccharide production [37]. Further functional groups comprise gene products that function in signal transduction (regulators and enzymes involved in production and turnover of secondary messenger molecules), however, none of these have been characterized in detail.

### UvrY mutants have distinct growth phenotypes

**Figure 5:**
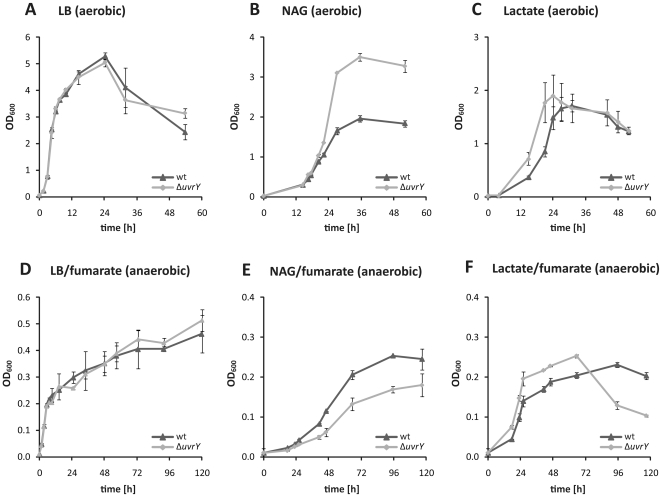
Aerobic and anaerobic growth of *S. oneidensis* wild type and *ΔuvrY* mutant in different media. The upper panel (A–C) displays growth under aerobic conditions, the lower panel (D–F) under anaerobic conditions. The corresponding electron donors and acceptors are indicated. Growth of the wild type is displayed in black, that of *ΔuvrY* in grey. In order to better highlight the differences in growth behavior, growth was plotted at a linear scale. The error bars display the standard deviation. NAG, N-acetylglucosamine.

A loss of BarA/UvrY and homologous regulation systems has not been linked to distinct growth phenotypes so far. In some species, mutants in BarA/UvrY have a temporary growth advantage and are dominant in long-term survival [38], [39], [40]. However, the occurrence of *ackA*, *aceAB*, and *pflAB* among the genes under positive control of UvrY in *S. oneidensis* MR-1 suggested a role of the BarA/UvrY two-component system in regulation of the central carbon metabolism in *S. oneidensis* MR-1. Therefore, we compared growth of a *ΔuvrY* mutant to that of the wild type under aerobic and anaerobic conditions with different carbon sources: complex LB medium and 4M mineral medium with either N-acetylglucosamine (NAG) or lactate as carbon source. In *S. oneidensis* MR-1, which is unable to grow on glucose, the glycolytic carbon source NAG is converted via the Entner-Doudoroff pathway while the gluconeogenic lactate enters the central metabolism at the stage of pyruvate [41], [42]. In complex LB media, no growth phenotype occurred under aerobic and anaerobic conditions (Fig. 5A,D). However, when the strains were grown in mineral medium with NAG as carbon source, the *ΔuvrY* mutants grew faster than the wild type (186±5.8 min vs. 233±8.8 min doubling time) and reached a significant higher OD_600_ (3.5±0.1 vs. 1.96±0.1). Notably, under anaerobic conditions with fumarate as terminal electron acceptor, the opposite effect was observed. The *ΔuvrY* mutants grew more slowly (1140±93 min vs. 1036±80 min) and reached a lower OD_600_ (0.18±0.03 vs. 0.25±0.01). With lactate as the carbon source, both strains grew at a similar rate, however, the *ΔuvrY* mutant entered the exponential growth phase earlier under both aerobic and anaerobic conditions. The OD_600_ reached by both strains was similar (1.9±0.4 vs. 1.67±0.27 under aerobic conditions; 0.23±0.01 vs. 0.25±0.01 under anaerobic conditions). To determine whether the increase in optical density could be attributed to cell numbers, we performed growth experiments with 1∶1 mixtures of wild-type and *ΔuvrY* mutants, in which one of two strains was constitutively expressing *gfp*. Cells were then enumerated by fluorescent microscopy at time points at which significant differences in OD were determined in single species cultures. An approximate 3∶2 ratio of *ΔuvrY*-mutant to wild-type cells occurred after 48 hours of aerobic growth with NAG (57.7±7.1% to 42.3±7.1%) and 24 hours with lactate (59.0±5.5% to 41±5.5%) as carbon source (Fig. S4B). Microscopic analysis did not reveal any significant differences with respect to cell morphologies, strongly indicating that the difference in optical density is at least in part due to the number rather than size or shape of the cells. A growth advantage of the *ΔuvrY* mutant over the wild type also occurred with acetate under aerobic conditions (data not shown). These growth characteristics suggested that BarA/UvrY might be involved in regulation of metabolism of both glycolytic or gluconeogenic carbon sources. To further determine whether this regulation is mediated through the sRNAs *csrB1* and *csrB2*, we performed aerobic growth experiments with NAG or lactate using a *ΔcsrB1ΔcsrB2* mutant. The growth characteristics of the *ΔcsrB1ΔcsrB2* mutant equaled those of *ΔuvrY* mutant (Fig. S4A), indicating the existence of a BarA/UvrY/Csr regulatory pathway in *Shewanella*.

### Activity of the GacA/UvrY system during growth on different carbon sources

**Figure 6:**
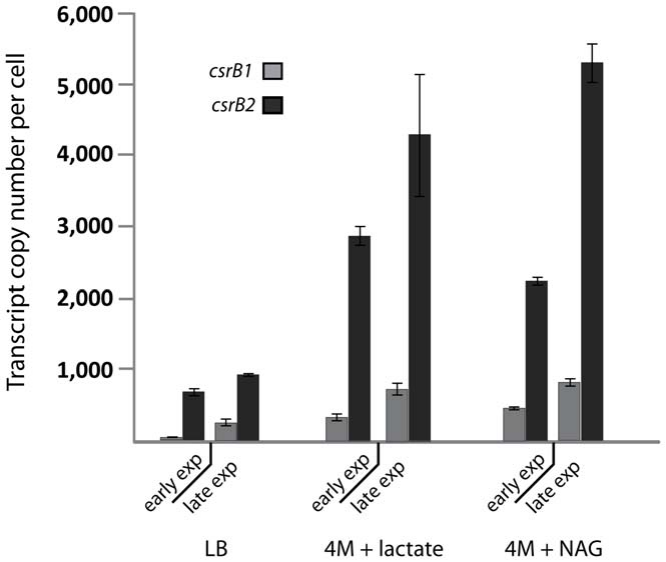
Expression levels of csrB1 and csrB2 system during growth with different carbon sources. Total RNA was prepared from cells growing in different media at early and late exponential phase as indicated below. The total RNA was then used to calculate the number of *csrB1* and *csrB2* transcripts by q-RT-PCR. NAG, N-acetylglucosamine.

The observed growth phenotypes in *S. oneidensis* MR-1 *ΔuvrY* mutants suggested that BarA/UvrY activity and expression of *csrB1* and *csrB2* depends on the carbon source. To determine the expression levels of the two sRNAs we performed q-RT-PCR on total RNA that was prepared during early and late exponential growth phase of wild-type cells grown in LB and 4M mineral medium with NAG or lactate as carbon source (Fig. 6). Under all conditions, expression of *csrB2* exceeded that of *csrB1* by a factor of about 10. Compared to growth in LB medium, the expression levels of both sRNAs was increased by a factor of 3 to 4 during growth in mineral medium, as has been recently observed in *E. coli* [43]. During late exponential phase the expression levels of the sRNAs increased with both lactate (1.3-fold) and NAG (2-fold) as carbon sources. As expected, in a *ΔuvrY* mutant expression of *csrB1* and *csrB2* dropped to low levels under all conditions (data not shown). Thus, in *S. oneidensis* MR-1, activity of the BarA/UvrY two-component system is directly or indirectly dependent on the carbon source and might be controlled by concentrations of intermediates or end products of central metabolism.

## Discussion

In this study, we have provided evidence that *S. oneidensis* MR-1 possesses a BarA/UvrY/Csr regulatory pathway. The corresponding *Shewanella* orthologs were identified by bioinformatic approaches, and we applied *in vitro* phosphotransfer studies to determine functional interactions between sensor kinase SO_3457 (now BarA) and and response regulator (now UvrY). We demonstrated that, under *in vitro* conditions, BarA readily autophosphorylates and transfers the phosporyl group to UvrY. As expected, no phosphotransfer was observed when the predicted phosphorylation site, aspartate residue 54, was substituted. However, under these conditions, a lower level of phosphorylated BarA occurred. Since the protein levels were not affected, this indicates a loss of the phosphate from BarA and suggests that in the presence of the mutated response regulator UvrY(D54N) the phosphorylation of the sensor kinase might be destabilized. To our knowledge, this has not been observed before. In concert with genetic studies, the *in vitro* phosphotransfer studies showed that SO_3457/BarA is the cognate sensor kinase for SO_1860/UvrY. We also demonstrated that in *S. oneidensis* MR-1, UvrY positively regulates at least two sRNAs, *csrB1* and *csrB2*. Also the corresponding CsrA ortholog can be readily identified in *Shewanella*, so that all major components of the regulatory pathway are present.

BarA/UvrY/Csr and orthologous pathways have been implicated in regulating global carbon metabolism, secondary metabolism, exoproduct formation, and pathogenicity in numerous species of the γ-proteobacteria [19]. To determine the corresponding regulon in *Shewanella*, we performed global transcriptomic analysis on a *uvrY* mutant. Recently, the transcriptome of a *Pseudomonas fluorescens* PF-5 *ΔgacA* mutant was analyzed under similar conditions [44]. 635 genes had different transcript levels in the mutant at late exponential growth phase. Thus, more than 10% of the annotated genes are under direct or indirect control of the UvrY ortholog GacA in this species. The most pronounced changes occurred in the regulation of genes involved in iron acquisition and homeostasis, TonB-signaling and ECF sigma factors, and secondary metabolism and exoenzymes. In *P. aeruginosa*, the expression of about 240 genes was affected in the absence of GacA or the corresponding sRNAs RsmYZ [25]. The study revealed that, in this species, genes encoding virulence factors, secretion systems and type IV pili are controlled by GacS/GacA/Rsm, the system orthologous to BarA/UvrY/Csr. These findings underline the crucial role of this regulatory cascade in secondary metabolism and pathogenicity in *Pseudomonas*. A similar study was carried out on a *uvrY* mutant of the insect pathogen *Photorhabdus luminescens* [45]. In this species, UvrY directly and indirectly controls the expression of more than 500 genes involved in flagellum synthesis, synthesis of the quorum-sensing autoinducer AI-2, iron transport, resistance, antibiotic synthesis, degradation, and virulence. It is thought that UvrY plays in important role in adaptation of *P. luminescens* inside the insect as a *uvrY* mutant is negatively affected in killing of insect cells and exhibits a reduced growth on insect cells cultures [45]. In contrast to Pseudomonads or *Photorhabdus luminescens*, a rather small set of genes is regulated by UvrY in the plant pathogen *Xylella fastidiosa*, comprising just 27 genes [46]. However, several among these genes encode important factors required to elicit disease symptoms on grapevines.

Thus, with the exception of two putative toxin transport systems (SOA_0047 – SOA_0050; SO_4318) that are potential virulence factors, UvrY controls a different set of genes in the *S. oneidensis* MR-1 compared to those of pathogenic species. Our transcriptome analysis revealed that among the genes controlled by this pathway, some encode key enzymes of global carbon metabolism. Similar regulation patterns to those observed in *S. oneidensis* MR-1 have been reported for non-pathogenic strains of *E. coli*, where genes involved in gluconeogenesis, glycolysis, pentose-phosphate and glyoxylate shunts, tricarboxylic acid (TCA) cycle, and the biosynthesis of glycogen and the exopolysaccharide poly-β-1,6-N-acetyl-D-glucosamine are regulated by the BarA/UvrY/Csr system [47], [48], [49], [50], [51]. In addition, BarA/UvrY mediates efficient switching between glycolytic and gluconeogenic carbon sources in *E. coli* [40]. Notably, few genes display striking changes in transcription levels in *S. oneidensis* MR-1 *uvrY* mutants. It has been hypothesized that, as opposed to pathogens such as *Pseudomonas*, *Photorhabdus*, or *Xyllela* species, in non-pathogenic *E. coli* strains the BarA/UvrY/Csr system rather has a modulating than a more decisive regulating role [19]. Our findings for *S. oneidensis* MR-1 support this hypothesis.

BarA/UvrY/Csr and orthologous systems have previously been demonstrated to regulate primary and secondary metabolism. However, our present study is, to our knowledge, the first report that directly links the BarA/UvrY two-component system to pronounced growth phenotypes in dependence of the carbon source. Thus, our present study implicates that, in *S. oneidensis* MR-1, the impact of BarA/UvrY on the control of central carbon fluxes is more pronounced than in other species examined so far. If BarA/UvrY plays a similar role in regulating secondary metabolism as demonstrated in other species such as *E. coli* or *Pseudomonas* sp., it is conceivable that, in *S. oneidensis* MR-1, BarA/UvrY controls the conversion of glycolytic NAG into storage compounds or cell material, such as exopolysaccharides. Accordingly, our transcriptomic analysis has identified several glycosyl transferases that are under positive control of UvrY in *S. oneidensis* MR-1. In addition, this species possess genes that are annotated to be involved in glycogen metabolism [5], [52]. Thus, we hypothesized that, during growth on NAG, wild-type cells might produce glycogen or exopolysaccharides while the *uvrY* mutant might use this carbon source exclusively for growth. Staining by iodine, congo red, or calcofluor white did not identify a difference between the wild type and a *uvrY* mutant (data not shown). Thus, potential exopolysaccharides and/or storage compounds are yet to be identified. Under anaerobic conditions, *S. oneidensis* MR-1 oxidizes N-acetylglucosamine and lactate to carbon dioxide and acetate as the main product, and energy conservation primarily occurs by substrate-level phosphorylation [1], [36], [53], [54], [55]. Our transcriptomic studies indicate that a key enzyme in this process, the acetate kinase AckA [36], is regulated by UvrY. It remains to be demonstrated whether growth phenotypes under anaerobic conditions may be attributed to differences in expression levels of *ackA* or genes encoding other enzymes of carbon metabolism.

The direct signal perceived by BarA/UvrY/Csr and orthologous pathways is still obscure. Expression of the pathways' sRNAs was observed to increase with cell density in a number of species in complex media [33], [56], [57], [58]. However, only *csrB1* was observed to display changing expression levels depending of the growth phase in complex medium, while *csrB2* is constantly expressed at a similar level independent of the growth phase. This indicates that *crsB1* and *csrB2* may be regulated differently in *S. oneidensis* MR-1. In contrast to other *Shewanella* species, such as *S. putrefaciens* CN-32, the two sRNAs are not adjacent but are separated by a transposase which might influence their expression. For *Pseudomonas* species it was demonstrated recently that other regulators, such as PsrA, IHF, MvaT, and MvaU, influence the expression in concert with the UvrY homolog GacA [24], [25]. In *Erwinia carotovora*, *rsmB*(*csrB*) expression is under negative control of RsmC, KdgR, and *hexR* [59], [60], [61]. Thus, it is conceivable that the expression of *csrB1* and *csrB2* is under direct control of other regulators in *S. oneidensis* MR-1 as well. In addition, studies on *P. fluorescens* revealed a strong positive correlation between expression of the Rsm sRNAs and pools of the TCA cycle intermediates 2-oxoglutarate, succinate, and fumarate [20]. If similar stimuli are responsible for the activation of the *Shewanella* BarA/UvrY system, the expression levels of *csrB1* and *csrB2* would be expected to be significantly different during growth with glycolytic NAG and gluconeogenetic lactate as carbon sources. However, this was not observed. Recent studies on the corresponding *E. coli* system suggested that formate and acetate primarily act as stimuli [21], and these compounds are also candidates to activate the BarA/UvrY system in *Shewanella*.

Notably, it is yet unclear how species of the genus *Shewanella* regulate the selection of carbon sources and carbon flux. Based on this study, it may be hypothesized that, in *Shewanella*, BarA/UvrY has adopted this role. All major components of the two-component system and downstream regulatory units can be readily identified in other *Shewanella* species. Thus, this study provides the basis for numerous future studies. Further mutant characterization, also including CsrA and the putative sRNAs CsrB1 and CsrB2, combined with carbon flux analysis and determination of enzyme activities will help to better understand the impact of the BarA/UvrY/Csr regulatory system on the global metabolism of *S. oneidensis* MR-1 and other *Shewanella* species.

## Materials and Methods

### Growth conditions and media

Bacterial strains used in this study are summarized in Table 1. *Escherichia coli* strains were routinely grown in LB medium at 37°C. For strain WM3064, 2,6-diamino-pimelic acid (DAP) was added to the medium to a final concentration of 300 µM. *Shewanella oneidensis* strains were routinely grown at 30°C in LB. For solidification, agar was added to a final concentration of 1.5% (w/v). Growth experiments were carried out in 4M mineral medium [62] supplemented with 40 mM N-acetylglucosamine (NAG) or 40 mM lactate as carbon sources with orbital shaking and repeated at least three independent times. Anaerobic growth was assayed in LB or in 4M mineral medium with 40 mM of the appropriate carbon source. 40 mM fumarate was added as terminal electron acceptor. To remove oxygen from media, the culture tubes were stoppered, sealed, and flushed with nitrogen gas for several minutes with periodic shaking [63]. Precultures were grown to late exponential phase in 20 ml of the corresponding media (LB, 4M-NAG or 4M-lactate). Appropriate volumes of the precultures were used for inoculation of the main cultures (50 ml) to an OD600 of 0.05. Where necessary, media were supplemented with 50 µg·ml^−1^ kanamycin sulfate, 10 µg·ml^−1^ chloramphenicol, and/or 100 µg·ml^−1^ ampicillin sodium salt.

**Table 1:** Strains and plasmids used in this study.

Strain or plasmid	Relevant genotype or description	reference
Bacterial strains		
*Escherichia coli*		
*E. coli* DH5αλpir	*recA1 gyrA* (*lacIZYA-argF*) (80d *lac* [*lacZ*] M15) *pir RK6*	[73]
*E. coli* WM3064	Donor strain for conjugation with *S. oneidensis* MR-1; *thrB1004 pro thi rpsL hsdS lacZ* M15 RP4-1360 (*araBAD*)*567 dapA1341*::[*erm pir*(wt)]	W. Metcalf, University of Illinois, Urbana-Champaign
*E. coli* BTH101a	F_ *cya-99 araD139 galE15 galK16 rpsL1* (Str^r^) *hsdR2 mcrA mcrB1*	Euromedex, France
*S. oneidensis*		
MR-1	Wild type	[74]
ΔSO_3457	in-frame deletion of SO_3457 (*barA*) in *S. oneidensis* MR-1	this study
ΔSO_1860	in-frame deletion of SO_1860 (*uvrY*) in *S. oneidensis* MR-1	this study
ΔSO_3457 ΔSO_1860	in-frame deletion of SO_3457 (*barA*) and SO_1860 (*uvrY*) in *S. oneidensis* MR-1	this study
ΔcrsB1ΔcsrB2	in-frame deletion of the gene region between SO_1617 and SO_1619 encoding CsrB1 and CsrB2 separated by SO_1616 encoding a transposase	
S 198	MR-1, tagged with eGfp in a mini-Tn*7* construct, Cm^r^	[66]
ΔSO_1860 gfp	ΔSO_1860, tagged with eGfp in a mini-Tn*7* construct, Cm^r^	this study
Plasmids		
Construction of in-frame deletions		
pNPTS138-R6KT	*mobRP4*_ *ori*-R6K *sacB*; suicide plasmid for in-frame deletions; Km^r^	[13]
pNPTS138-R6KT-ΔSO_3457	SO_3457 (*barA*) deletion fragment in pNPTS138-R6KT	this study
pNPTS138-R6KT-ΔSO_1860	SO_1860 (*uvrY*) deletion fragment in pNPTS138-R6KT	this study
pTNS2	ori-R6K; encodes the TnsABC+D specific transposition pathway, Ap^r^	[75]
pUC18-R6KT-miniTn7T-egfp	MiniTn*7*T-based system for construction of strains constitutively expressing *gfp*, Ap^r^, Cm^r^	[66]
Overexpression of SO_3457 (*barA*) and SO_1860 (*uvrY*)		
pBAD-HisA	Over-expression vector; L-arabinose promoter; N-terminal 6xhistidine fusion tag; Amp^r^	Invitrogen, Frankfurt, Germany
pGEX-4T1	Over-expression vector; lactose promoter; N-terminal GST fusion tag; Amp^r^	GE Healthcare, München, Germany
pBAD-HisA-SO_3457	SO_3457 (*barA*) C-terminal coding region (aa 181–929) in pBAD-HisA resulting in N-terminal 6xHis fusion	this study
pGEX-4T1-SO_1860	SO_1860 (*uvrY*) in pGEX-4T1 resulting in N-terminal GST fusion	this study
pGEX-4T1-SO_1860_D54N	SO_1860 (*uvrY*) containing point mutation (G-A; bp 161) in pGEX-4T1 resulting in protein with D54N mutation and N-terminal GST fusion	this study

Amp^r^, ampicillin resistance; Cm^r^, chloramphenicol resistance; Km^r^, kanamycin resistance.

To determine cell numbers during growth of *ΔuvrY* cells compared to those of the wild type, mixed cultures containing 1∶1 ratios of wild type and mutant were used. Precultures of the wild type and *ΔuvrY* mutant were grown to late exponential phase in 4M-medium containing 40 mM NAG or 40 mM lactate. To distinguish the cell type, one of the strains in the cultures was constitutively expressing *gfp*. To exclude that *gfp* expression affects growth rate and cell number, we determined the cell numbers from two different mixtures (wt-gfp/*ΔuvrY* or wt/*ΔuvrY*-gfp). The ratio of the corresponding strains was determined in the beginning to ensure a 1∶1 ratio and at appropriate time points by fluorescent microscopy using a using an upright Zeiss Image MI (Oberkochen, Germany) equipped with Cascade 1 K camera (Visitron Systems, Puchheim, Germany) and a Zeiss Plan Apochromat 100x/1.4 objective. At least 180 cells per slide were counted.

### Vector and strain constructions

DNA manipulations were performed according to standard protocols [64], [65] or following the manufacturers' instructions. Kits for the isolation of chromosomal DNA, the isolation of plasmids and the purification of polymerase chain reaction (PCR) products were purchased from HISS Diagnostics GmbH (Freiburg, Germany). Enzymes were purchased from New England Biolabs (Frankfurt, Germany) and Fermentas (St Leon-Rot, Germany). Strains and plasmids used in this study are summarized in Table 1. Sequencing of DNA was carried out at Eurofins MWG GmbH, Ebersberg, Germany.

Markerless in-frame deletion mutants of *S. oneidensis* MR-1 were constructed essentially as reported earlier [13] using the suicide vector pNPTS-138-R6KT and appropriate primer pairs (Table S4). For confirmation of phenotypes induced by in-frame deletions, the *S. oneidensis* MR-1 genotype was restored by replacing the in-frame deletion by the wild-type copy of the gene following the deletion strategy.

A ΔSO_1860/*ΔuvrY* strain constitutively expressing *gfp* was constructed by using a modified Tn*7* delivery system as reported earlier [66]. Briefly, the plasmid pUC18-R6KT-miniTn7T-egfp was used for tagging *S. oneidensis ΔuvrY* by three-parental mating from the DAP-auxotroph *E. coli* WM3064 and *E. coli* WM3064 harboring the helper plasmid pTNS2.

Genes and gene fragments to be overexpressed were amplified from template genomic DNA using appropriate primers (Tab. S1). Site-directed mutagenesis (D_54_N) in SO_1860/*uvrY* was achieved using overlap extension PCR as previously described [67]. The PCR fragment harboring *barA*_181–929_ was ligated into pBAD-HisA (Invitrogen) to result in a N-terminal His-tag fusion, *uvrY* and *uvrY*(D_54_N) were cloned into pGEX4T-1 (GE-Healthcare) yielding N-terminal fusions to glutathione S-transferase (GST).

### RNA extraction from *S. oneidensis* MR-1

Cells at the appropriate growth phase were harvested by centrifugation at 4600×g for 15 min at 4°C, and the cell sediments were immediately frozen in liquid nitrogen and stored at −80°C. Total RNA was extracted from *S. oneidensis* MR-1 cells using the hot phenol method [68]. Residual chromosomal DNA was removed using Turbo DNA-*free*™ (Applied Biosystems) following the manufacturer's instructions. The purified RNA was then used for transcriptomic profiling by microarray analysis, quantitative Real Time PCR (q-RT PCR), and Northern analysis.

### Northern analysis of *csrB1* and *csrB2* transcripts

15 µg total RNA extracted from the *S. oneidensis* wild type strain at late exponential growth phase (OD_600_ 4.0) was separated in denaturating gel electrophoresis and transferred to an Amersham Hybond™-N^+^ nylon membrane (GE Healthcare, Freiburg, Germany). Nucleic acids were covalently bound to the membrane by UV-cross linking at 254 nm for 3 minutes. For the detection of *csrB1* and *csrB2* transcripts digoxygenin (DIG)-labeled DNA probes were synthesized using the PCR DIG Probe Synthesis Kit (Roche, Mannheim, Germany). Hybridization was carried out using the DIG Nucleic Acid Detection Kit (Roche Mannheim) according to the manufacturer's instructions. Signals were detected using the CDP-Star® reagent (New England Biolabs, Frankfurt am Main, Germany) and documented using an Immunoblot Imager LAS 4000 (FujiFilm, Düsseldorf, Germany).

### Transcriptomic analysis

Microarray analysis was performed by febit biomed GmbH (Heidelberg, Germany) using arrays containing three 50mer probes for each annotated gene of *S. oneidensis* MR-1. For each strain, three independent RNA samples obtained from three independent experiments were analyzed. Array design, handling, expression profiling, and data analysis has been described in detail previously [13]. The raw data and normalized data are available under Gene Expression Omnibus under accession GSE24994. After verification of the normal distribution of the measured data, parametric t-test (unpaired, two-tailed) was carried out for each gene separately, to detect genes that show a differential expression between the compared groups. The resulting P-values were adjusted for multiple testing by Benjamini-Hochberg adjustment [69], [70]. For significant statistical measurements, an adjusted P-value<0.05 (5%) cut-off was applied. All data obtained is MIAME compliant.

### q-RT-PCR

Extracted total RNA was applied as template for random-primed First Strand cDNA Synthesis using Bioscript™ (Bioline) following the manufacturer's instructions. The cDNA was used as template for quantitative RT-PCR (Real Time 7300 PCR Machine, Applied Biosystems) using the Sybr Green detection system (Applied Biosystems). The signals were standardized to *recA*, with the CT (cycle threshold) determined automatically by the Real Time 7300 PCR software (Applied Biosystems), and the total number of cycles was set to 40. Samples were assayed at least in duplicate. The efficiency of each primer pair was determined using four different concentrations of *S. oneidensis* MR-1 chromosomal DNA (10 ng·l^−1^, 1.0 ng·l^−1^, 0.1 ng·l^−1^, and 0.01 ng·l^−1^) as a template in quantitative PCRs.

### Overproduction and purification of recombinant proteins

*E. coli* strains carrying the corresponding protein expression plasmids (either pBAD-HisA or pGEX4T1) were grown overnight with orbital shaking (200 rpm) in SOB medium. The cultures were then used for reinoculation of 500 ml SOB medium at an OD_600_ of 0.1. At an OD_600_ of 0.5, expression from pBAD-HisA constructs was induced by the addition of L-arabinose to a final concentration of 0.2% (w/v) and expression from pGEX4T1 constructs by the addition of isopropyl-1-thio-β-D-galactopyranoside (IPTG) to a final concentration of 1 mM. Subsequently, the cells were incubated for 4 h at 37°C.

For purification of recombinant proteins, cells were resuspended in lysis buffer (50 mM NaH_2_PO_4_ pH 8.0, 300 mM NaCl, 0.2 mM phenylmethanesulfonylfluoride (PMSF), 0.5 mg/ml lysozyme) and lysed by three passages through a French Press (SLM-AMINCO/Spectronic) at 1240 bar (18,000 psi). The lysate was centrifuged at 35,000×*g* for 1 h at 4°C, and the supernatant was filtered (0.45 µm). The purification was performed by affinity chromatography at 4°C following the manufacturer's instructions in a batch procedure using either 1 ml Ni-NTA Superflow (Qiagen, Hilden, Germany) for His_6_-Proteins or 1 ml GST-Bind™ resin (Novagen) for GST fusion proteins. Elution fractions containing purified protein were pooled and dialyzed overnight at 4°C against TGMNKD buffer (50 mM Tris-HCl, pH 8.0, 10% (v/v) glycerol, 5 mM MgCl_2_, 150 mM NaCl, 50 mm KCl, 1 mM dithiothreitol) prior to use for further assays. The total protein concentration was determined via the Bio-Rad Protein assay (Bio-Rad Labratories GmbH, Munich, Germany) following the manufacturer's instructions.

### Radiolabeled *in vitro* autophosphorylation and phosphotransfer assays

*In vitro* phosphorylation BarA was carried out in TGMNKD buffer containing 0.5 mM [γ^32P^]ATP (14.8 GBq mmol^−1^; Amersham) and 10 µM of the corresponding protein in 50 µl total volume for 30 min at room temperature. Aliquots of 10 µl were quenched with 2 µl of 5× Laemmli sample buffer (0.313 M Tris-HCl (pH 6.8 at 25°C), 10% SDS, 0.05% bromophenol blue and 50% glycerol) [71], [72]. Phosphotransfer reactions with purified BarA, UvrY, and UvrY_D54N_ were performed by first autophosphorylating 10 µM BarA for 5 min as above. An aliquot was removed for an autophosphorylation control. An equivalent volume containing response regulator at equal concentration was then added to the reaction and incubated for 1 min. Both reactions were then quenched with 5× Laemmli sample buffer (kinase final concentration: 2.5 µM).

To determine autophosphorylation of UvrY by acetyl phosphate, 10 µM of the protein were incubated in TGMNKD buffer with an equivalent volume of acetyl [^32^P]-phosphate for 10, 30, 60, and 120 min at room temperature [72]. The reaction was quenched with 5× Laemmli sample buffer (0.125 M Tris-HCl, pH 6.8, 20% glycerol, 4% SDS, 10% β-mercaptoethanol, 0.02% bromphenol blue). Radioactive acetyl phosphate was generated by incubating the following reaction: 1.5 units of acetate kinase (Sigma), TKM buffer (2.5 mM Tris-HCl, pH 7.6, 6 mM potassium acetate, 1 mM MgCl_2_), and 10 µl of [γ-^32^P]ATP (14.8 GBq·mmol^−1^; Amersham Biosciences) in a total volume of 100 µl. The reaction was incubated for 2 h at room temperature. To remove the acetate kinase, the reaction was subjected to centrifugation in a Microcon YM-10 centrifugal filter unit (Millipore) for 1 h. The flow-through was collected and stored at 4°C.

For analysis of the autophosphorylation or the phosphotransfer reaction, 10 µl samples were loaded without prior heating on a 15% polyacrylamide gel, and separated by denaturating sodium dodecyl sulfate polyacrylamide gel electrophoresis (SDS-PAGE). Subsequently, gels were exposed to a PhosphorImager screen overnight, and images were detected on a Typhoon Trio PhosphorImager (Amersham Biosciences, GE Healthcare, Freiburg, Germany). Gels were subsequently stained by Coomassie dye (Carl Roth, Karlsruhe, Germany) to visualize proteins.

## Supporting Information

Figure S1 ***in vitro*** **interaction of BarA and UvrY.** A) Autoradiographic analysis of BarA phosphorylation. Upper panel: autoradiographic image, lower panel: corresponding PAGE after Coomassie staining. B) Upper panel: Autoradiographic analysis of phosphotransfer between BarA(181–929) and UvrY. The idicated components were added to the reaction. Lower panel: corresponding PAGE after Coomassie staining.
(PDF)

Figure S2 **Secondary structures of putative sRNAs CsrB1 and CsrB2.** The corresponding sequences as predicted by Kulkarni et al. (2006) were used for secondary structure prediction by CentroidFold (www.ncrna.org/centroidfold; Sato et al., 2009). The color panel displays the probability of base pairing from 0 (blue) to 1 (red). Kulkarni, P. R., X. Cui, J. W. Williams, A. M. Stevens, and R. V. Kulkarni. 2006. Prediction of CsrA-regulating small RNAs in bacteria and their experimental verification in *Vibrio fischerii*. Nucleic Acids Res. **34**:3361–3369. Sato, K., M. Hamada, K., Asai, and T. Mituyama. 2009. CENTROIDFOLD: a web server for RNA secondary structure prediction. Nucleic Acids Res. **37**:W277–280.
(PDF)

Figure S3 **Comparison of transcriptional changes in** ***ΔuvrY*** **mutants as determined by microarrays and q-RT-PCR.** Values of transcriptional level changes (log2) of 5 selected genes were plotted next to (A) and against each other (B). The gene identities are indicated in A.
(PDF)

Figure S4 A) Growth comparison of *S.oneidensis* MR-1 wild type, *ΔuvrY*, and *ΔcsrB1ΔcsrB2* mutants. Displayed is growth under aerobic conditions with N-acetylglucosamine (NAG) as carbon source (upper panel) or lactate (lower panel). The corresponding strains are indicated. In order to better highlight the differences in growth behaviour, growth was plotted at a linear scale. The error bars display the standard deviation. B) Ratio of *ΔuvrY* and wild-type cells grown in mixed cultures. To distinguish the two strains, one of the strains was constitutively expressing *gfp*.
(PDF)

Table S1 **Putative orthologs of BarA and UvrY in** ***Shewanella*** **species.**
(PDF)

Table S2 **Significantly upregulated genes in Δ** ***uvrY*** **.**
(PDF)

Table S3 **Significantly downregulated genes in Δ** ***uvrY*** **.**
(PDF)

Table S4 **Primer used in this study.**
(PDF)
