# Supplementary material for: Analysis of the BarA/UvrY Two-Component System in Shewanella oneidensis MR-1
Source: PLoS One. 2011 Sep 12;6(9):e23440. doi: 10.1371/journal.pone.0023440 (PMC3171408; doi:10.1371/journal.pone.0023440)
Supplement: Figure S1 — in vitro interaction of BarA and UvrY. A) Autoradiographic analysis of BarA phosphorylation. Upper panel: autoradiographic image, lower panel: corresponding PAGE after Coomassie staining. B) Upper panel: Autoradiographic analysis of phosphotransfer between BarA(181–929) and UvrY. The idicated components were added to the reaction. Lower panel: corresponding PAGE after Coomassie staining. (PDF) [file pone.0023440.s001.pdf]

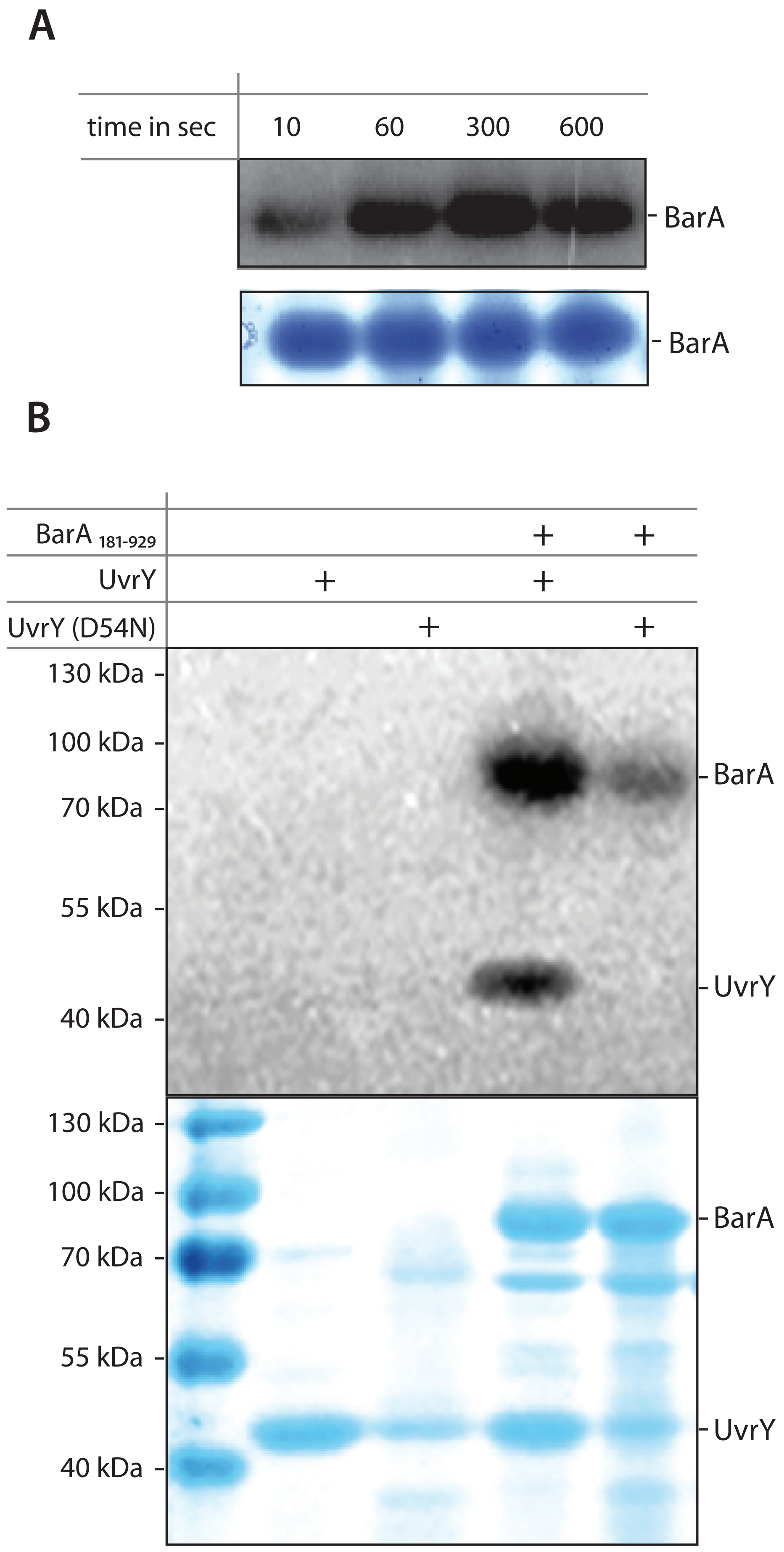

Figure S1: in vitro interaction of BarA and UvrY. A) Autoradiographic analysis of BarA phosphorylation. Upper panel: autoradiographic image, lower panel: corresponding PAGE after Coomassie staining. B) Upper panel: Autoradiographic analysis of phosphotransfer between BarA(181-929) and UvrY. The idicated components were added to the reaction. Lower panel: corresponding PAGE after Coomassie staining.
