# Supplementary material for: Analysis of the BarA/UvrY Two-Component System in Shewanella oneidensis MR-1
Source: PLoS One. 2011 Sep 12;6(9):e23440. doi: 10.1371/journal.pone.0023440 (PMC3171408; doi:10.1371/journal.pone.0023440)
Supplement: Figure S2 — Secondary structures of putative sRNAs CsrB1 and CsrB2. The corresponding sequences as predicted by Kulkarni et al. (2006) were used for secondary structure prediction by CentroidFold (www.ncrna.org/centroidfold; Sato et al., 2009). The color panel displays the probability of base pairing from 0 (blue) to 1 (red). Kulkarni, P. R., X. Cui, J. W. Williams, A. M. Stevens, and R. V. Kulkarni. 2006. Prediction of CsrA-regulating small RNAs in bacteria and their experimental verification in Vibrio fischerii. Nucleic Acids Res. 34:3361–3369. Sato, K., M. Hamada, K., Asai, and T. Mituyama. 2009. CENTROIDFOLD: a web server for RNA secondary structure prediction. Nucleic Acids Res. 37:W277–280. (PDF) [file pone.0023440.s002.pdf]

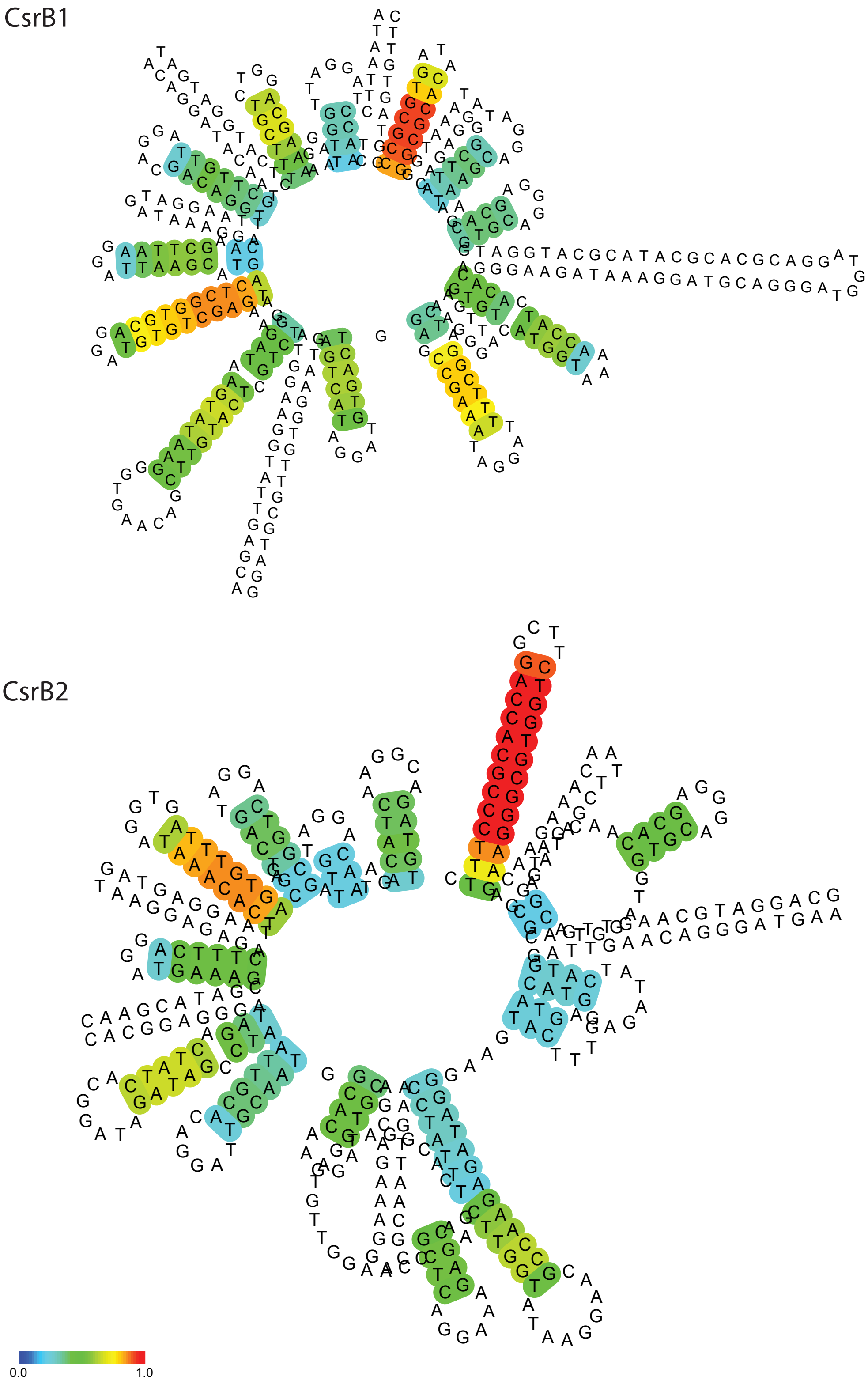

Figure S2: Secondary structures of putative sRNAs CsrB1 and CsrB2. The corresponding sequences as predicted by Kulkarni et al. (2006) were used for secondary structure prediction by CentroidFold (www.ncrna.org/centroidfold; Sato et al., 2009). The color panel displays the probability of base pairing from 0 (blue) to 1 (red).

Kulkarni, P. R., X. Cui, J. W. Williams, A. M. Stevens, and R. V. Kulkarni. 2006. Prediction of CsrA-regulating small RNAs in bacteria and their experimental verification in *Vibrio fischerii*. Nucleic Acids Res. **34**:3361-3369

Sato, K., M. Hamada, K., Asai, and T. Mituyama. 2009. CENTROIDFOLD: a web server for RNA secondary structure prediction. Nucleic Acids Res. **37**:W277-280.
