# Supplementary material for: Analysis of the BarA/UvrY Two-Component System in Shewanella oneidensis MR-1
Source: PLoS One. 2011 Sep 12;6(9):e23440. doi: 10.1371/journal.pone.0023440 (PMC3171408; doi:10.1371/journal.pone.0023440)
Supplement: Figure S3 — Comparison of transcriptional changes in ΔuvrY mutants as determined by microarrays and q-RT-PCR. Values of transcriptional level changes (log2) of 5 selected genes were plotted next to (A) and against each other (B). The gene identities are indicated in A. (PDF) [file pone.0023440.s003.pdf]

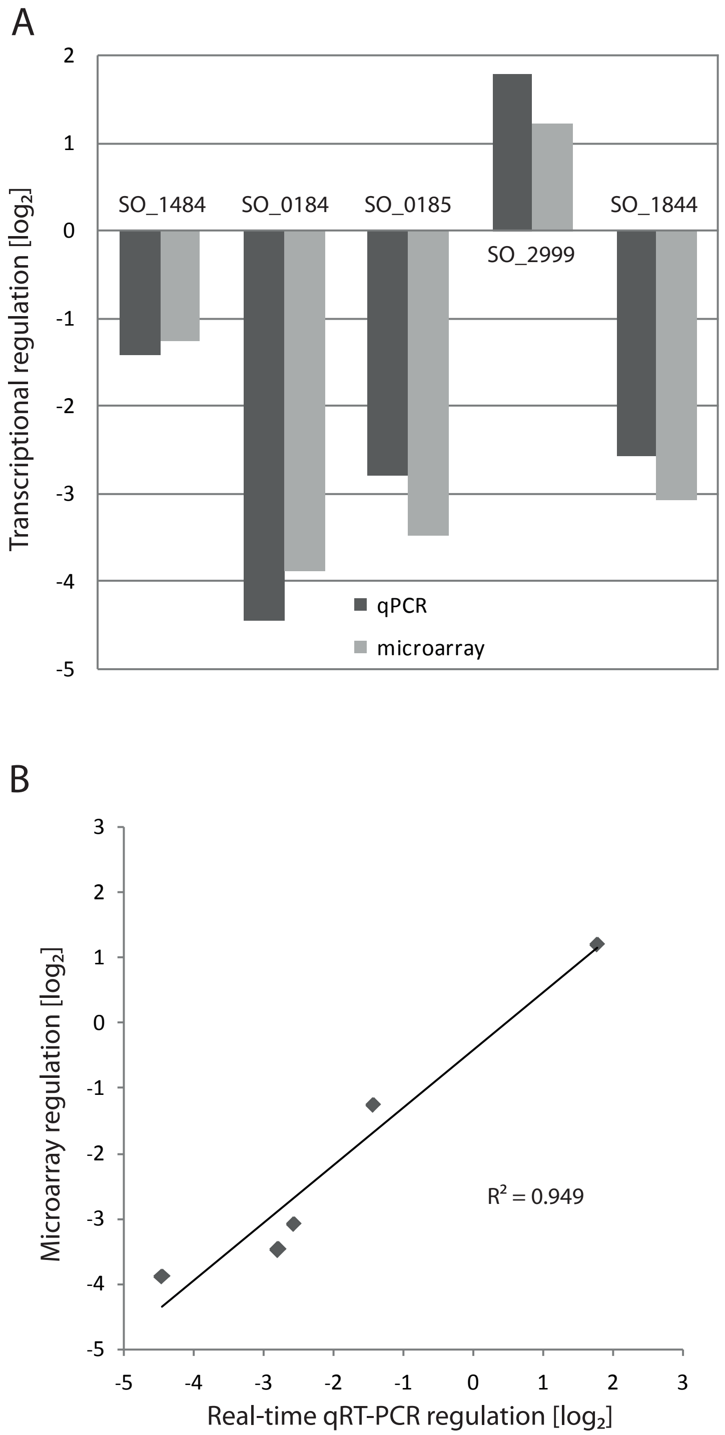

Figure S3: Comparison of transcriptional changes in ΔuvrY mutans as determined by microarrays and q-RT-PCR. Values of transcriptional changes (log2) of 5 selected genes were plotted next to (A) and against (B) each other. The gene identities are indicated in A.
