# Supplementary material for: Analysis of the BarA/UvrY Two-Component System in Shewanella oneidensis MR-1
Source: PLoS One. 2011 Sep 12;6(9):e23440. doi: 10.1371/journal.pone.0023440 (PMC3171408; doi:10.1371/journal.pone.0023440)
Supplement: Figure S4 — A) Growth comparison of S.oneidensis MR-1 wild type, ΔuvrY, and ΔcsrB1ΔcsrB2 mutants. Displayed is growth under aerobic conditions with N-acetylglucosamine (NAG) as carbon source (upper panel) or lactate (lower panel). The corresponding strains are indicated. In order to better highlight the differences in growth behaviour, growth was plotted at a linear scale. The error bars display the standard deviation. B) Ratio of ΔuvrY and wild-type cells grown in mixed cultures. To distinguish the two strains, one of the strains was constitutively expressing gfp. (PDF) [file pone.0023440.s004.pdf]

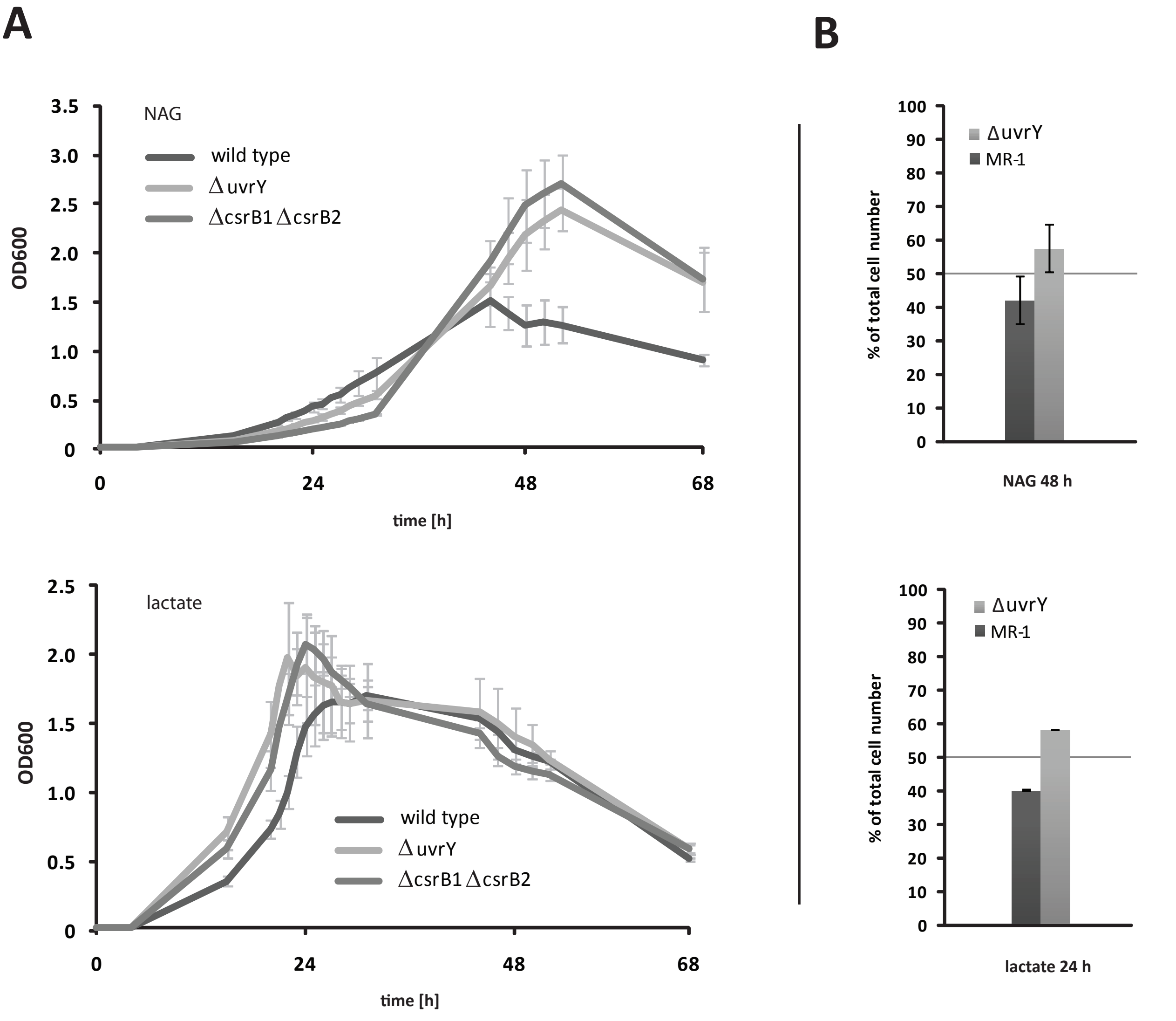

Figure S4: A) Growth comparison of *S. oneidensis* wild type, *ΔuvrY,* and *ΔcsrB1ΔcsrB2* mutants. Displayed is growth under aerobic conditions with N-acetylglucosamine (NAG) as carbon source (upper panel) or lacate (lower panel). The corresponding strains are indicated. In order to better highlight the differences in growth behavior, growth was plotted at a linear scale. The error bars display the standard deviation. **B)** Ratio of *ΔuvrY* and wild-type cells grown in mixed cultures. To distinguish the two strains, one of the strains was constitutively expressing *gfp*.
