# Supplementary material for: Analysis of the BarA/UvrY Two-Component System in Shewanella oneidensis MR-1
Source: PLoS One. 2011 Sep 12;6(9):e23440. doi: 10.1371/journal.pone.0023440 (PMC3171408; doi:10.1371/journal.pone.0023440)
Supplement: Table S1 — Putative orthologs of BarA and UvrY in Shewanella species. (PDF) [file pone.0023440.s005.pdf]

**Table S1**:  Putative orthologs of BarA and UvrY in *Shewanella* species.

| **Species** | **BarA** (% identity/similarity) | **UvrY** (% identity/similarity) |
|---|---|---|
| *Shewanella oneidensis* MR-1 | BarA/SO_3457 (100/100) | UvrY/SO_1860 (100/100) |
| *Shewanella* sp. MR-7 | Shewmr7_1175 (96/98) | Shewmr7_1614(99/99) |
| *Shewanella* sp. ANA-3 | Shewana3__1109 (96/98) | Shewana3__1608 (99/99) |
| *Shewanella* sp. MR-4 | Shewmr4_1175 (96/97) | Shewmr4_1547 (99/99) |
| *Shewanella* sp. W3-18-1 | Sputw3181_1245 (91/96) | Sputw3181_2386 (99/99) |
| *Shewanella putrefaciens* CN-32 | Sputcn32_2767 (91/96) | Sputcn32_1639 (99/99) |
| *Shewanella baltica* OS185 | Shew185_3146 (91/96) | Shew185_2600 (99/99) |
| *Shewanella baltica* OS223 | Sbal223_1223 (91/96) | Sbal223_1782 (99/99) |
| *Shewanella baltica* OS195 | Sbal195_3291 (91/96) | Sbal195_2677 (99/99) |
| *Shewanella baltica* OS183 | Sbal183_1636 (91/96) | Sbal183_0659 (99/99) |
| *Shewanella baltica* OS155 | Sbal_3148 (91/96) | Sbal_2562 (99/99) |
| *Shewanella frigidimarina* NCIMB 400 | Sfri_1047 (69/82) | Sfri_2649 (90/95) |
| *Shewanella denitrificans* OS217 | Sden_1191 (68/81) | Sden_1436 (89/94) |
| *Shewanella sediminis*  HAW-EB3 | Ssed_1285 (70/81) | Ssed_2455 (91/95) |
| *Shewanella amazonensis* SB2B | Sama_1031 (69/82) | Sama_1607 (84/92) |
| *Shewanella paleana* ATCC 700345 | Spea_1180 (69/81) | Spea_1953 (89/95) |
| *Shewanella piezotolerans* WP3 | Swp_1351 (68/81) | Swp_2799 (89/96) |
| *Shewanella halifaxensis* HAW-EB4 | Shai_1217 (68/80) | Shai_2346 (90/95) |
| *Shewanella violacea* DSS12 | SVI_3181 (67(80) | SVI_1867 (88/95) |
| *Shewanella woodyi* ATCC 51908 | Swoo_3355 (67/79) | Swoo_2150 (90/94) |
| *Shewanella loihica* PV-4 | Shew_1200 (65/78) | Shew_1824 (88/96) |
