# Supplementary material for: Analysis of the BarA/UvrY Two-Component System in Shewanella oneidensis MR-1
Source: PLoS One. 2011 Sep 12;6(9):e23440. doi: 10.1371/journal.pone.0023440 (PMC3171408; doi:10.1371/journal.pone.0023440)
Supplement: Table S2 — Significantly upregulated genes in Δ uvrY . (PDF) [file pone.0023440.s006.pdf]

Table S2: significantly upregulated genes in $\Delta uvrY$

ORF	Gene	Regulation[1]	Product	COG description
**SO_0095**	hutI	**1.26**	imidazolonepropionase	Secondary metabolites biosynthesis. transport and catabolism
**SO_0106**	selB	**1.07**	selenocysteine-specific translation elongation factor	Translation. ribosomal structure and biogenesis
**SO_0236**	rplV	**1.25**	50S ribosomal protein L22	Translation. ribosomal structure and biogenesis
**SO_0240**	rpsQ	**1.06**	ribosomal protein S17	Translation. ribosomal structure and biogenesis
**SO_0363**	-	**1.15**	hypothetical protein	Cell wall/membrane/envelope biogenesis. Translation. ribosomal structure and biogenesis
**SO_0425**	aceF	**1.18**	pyruvate dehydrogenase complex. E2 component. dihydrolipoamide acetyltransferase	Energy production and conversion
**SO_0552**	-	**3.15**	hypothetical protein	Function unknown
**SO_0562**	-	**1.21**	hypothetical protein	Function unknown
**SO_0704**	groEL	**1.50**	chaperonin GroEL	Posttranslational modification. protein turnover. chaperones
**SO_0730**	-	**1.41**	hypothetical protein	Function unknown
**SO_0753**	-	**1.28**	hypothetical protein	Function unknown
**SO_0755**	-	**2.09**	hypothetical protein	Function unknown
**SO_0921**	-	**1.35**	hypothetical protein	Function unknown
**SO_1011**	nuoL	**1.11**	NADH dehydrogenase subunit L	Energy production and conversion. Inorganic ion transport and metabolism
**SO_1121**	proB	**1.02**	gamma-glutamyl kinase	Amino acid transport and metabolism
**SO_1182**	-	**5.56**	hypothetical protein	Function unknown
**SO_1568**	-	**1.68**	hypothetical protein	Function unknown
**SO_1594**		**1.42**	binding-protein-dependent transport systems inner membrane component	
**SO_1672**	-	**1.54**	hypothetical protein	Function unknown
**SO_1744**	-	**1.36**	peptide synthase	Lipid transport and metabolism. Secondary metabolites biosynthesis. transport and catabolism
**SO_1808**	pspB	**1.20**	phage shock protein B	Function unknown
**SO_1817**	-	**1.15**	primosomal replication protein n''. putative	Replication. recombination and repair
**SO_1843**	-	**1.42**	hypothetical protein	Function unknown
**SO_1848**	-	**1.00**	hypothetical protein	Function unknown
**SO_1877**	bcp	**1.07**	thioredoxin-dependent thiol peroxidase	Posttranslational modification. protein turnover. chaperones
**SO_1970**	-	**1.07**	hypothetical protein	Function unknown
**SO_2007**	-	**1.14**	hypothetical protein	Function unknown
**SO_2111**	-	**1.17**	hypothetical protein	Function unknown
**SO_2389**	emrD	**1.19**	multidrug resistance protein D	Carbohydrate transport and metabolism. Amino acid transport and metabolism. Inorganic ion transport and metabolism. General function prediction only
**SO_2480**	-	**1.45**	hypothetical protein	Replication. recombination and repair
**SO_2545**	-	**1.18**	sensor histidine kinase	Signal transduction mechanisms
**SO_2653**	-	**2.09**	Ner family transcriptional regulator	Transcription
**SO_2654**	-	**1.92**	transposase. putative	Replication. recombination and repair
**SO_2656**	-	**4.10**	hypothetical protein	Function unknown

Table S2: significantly upregulated genes in $\Delta uvrY$

ORF	Gene	Regulation[1]	Product	COG description
**SO_2657**	-	**1.89**	hypothetical protein	Function unknown
**SO_2660**	-	**2.50**	hypothetical protein	Function unknown
**SO_2666**	-	**1.03**	hypothetical protein	Function unknown
**SO_2688**	-	**2.37**	hypothetical protein	Function unknown
**SO_2766**	-	**1.84**	hypothetical protein	General function prediction only
**SO_2808**	-	**1.55**	hypothetical protein	Cell cycle control. cell division. chromosome partitioning
**SO_2833**	nrdG	**1.05**	anaerobic ribonucleotide reductase-activating protein	Posttranslational modification. protein turnover. chaperones
**SO_2841**	-	**1.20**	hypothetical protein	Function unknown
**SO_2980**	-	**1.85**	hypothetical protein	Function unknown
**SO_2988**	-	**1.34**	hypothetical protein	Function unknown
**SO_2995**	-	**1.37**	hypothetical protein	Function unknown
**SO_2999**	-	**1.78**	hypothetical protein	Function unknown
**SO_3003**	-	**1.54**	hypothetical protein	Function unknown
**SO_3004**	-	**1.59**	prophage LambdaSo. DNA modification methyltransferase. putative	Function unknown
**SO_3132**	-	**2.32**	hypothetical protein	Function unknown
**SO_3226**	fliH	**1.17**	flagellar assembly protein H	Cell motility. Intracellular trafficking. secretion. and vesicular transport
**SO_3268**	-	**1.12**	alpha amylase domain-containing protein	Carbohydrate transport and metabolism
**SO_3305**	-	**1.31**	LuxR family DNA-binding response regulator	Signal transduction mechanisms. Transcription
**SO_3370**	-	**1.31**	hypothetical protein	Function unknown
**SO_3410**	-	**1.62**	hypothetical protein	Cell wall/membrane/envelope biogenesis
**SO_3442**	mazG	**1.61**	nucleoside triphosphate pyrophosphohydrolase	General function prediction only
**SO_3543**	-	**2.09**	ISSod13. transposase	Replication. recombination and repair
**SO_3642**	-	**1.29**	methyl-accepting chemotaxis protein	Cell motility. Signal transduction mechanisms
**SO_3725**	-	**1.02**	hypothetical protein	Function unknown
**SO_3743**	-	**1.09**	TetR family transcriptional regulator	Transcription
**SO_3777**	-	**1.00**	hypothetical protein	Replication. recombination and repair
**SO_3810**	-	**1.71**	OmpA-like transmembrane domain-containing protein	Cell wall/membrane/envelope biogenesis
**SO_3985**	-	**1.13**	hypothetical protein	General function prediction only
**SO_4014**	-	**1.17**	AcrB/AcrD/AcrF family protein	Defense mechanisms
**SO_4099**		**1.32**	phosphopentomutase (EC:5.4.2.7); K01839 phosphopentomutase [EC:5.4.2.7]	
**SO_4177**	-	**1.23**	hypothetical protein	Function unknown
**SO_4187**	-	**1.17**	hypothetical protein	Function unknown
**SO_4196**	-	**1.02**	hypothetical protein	Function unknown

Table S2: significantly upregulated genes in Δ*uvrY*

ORF	Gene	Regulation[1]	Product	COG description
**SO_4435**	-	**1.24**	hypothetical protein	Function unknown
**SO_4436**	-	**1.31**	ISSod11. transposase	Replication. recombination and repair
**SO_4452**	moaA	**1.35**	molybdenum cofactor biosynthesis protein A	Coenzyme transport and metabolism
**SO_4462**	-	**1.27**	hypothetical protein	Function unknown
**SO_4503**	-	**1.07**	formate dehydrogenase accessory protein FdhD. putative	Energy production and conversion
**SO_4557**	-	**1.37**	methyl-accepting chemotaxis protein	Cell motility. Signal transduction mechanisms
**SO_4695**		**1.23**	hypothetical protein	
**SO_4721**	-	**1.52**	ABC transporter. ATP-binding protein	Inorganic ion transport and metabolism
**SO_A0058**	-	**1.20**	hypothetical protein	Function unknown
**SO_A0069**	-	**1.10**	hypothetical protein	Function unknown
**SO_A0096**	parB	**1.39**	plasmid partition protein ParB	Transcription
**SO_A0115**	-	**1.26**	putative lipoprotein	Function unknown

[1]log fold change
