# Supplementary material for: Analysis of the BarA/UvrY Two-Component System in Shewanella oneidensis MR-1
Source: PLoS One. 2011 Sep 12;6(9):e23440. doi: 10.1371/journal.pone.0023440 (PMC3171408; doi:10.1371/journal.pone.0023440)
Supplement: Table S3 — Significantly downregulated genes in Δ uvrY . (PDF) [file pone.0023440.s007.pdf]

Table S3: significantly downregulated genes in Δ*uvrY*

ORF	Gene	Regulation[1]	Product	COG description
**SO_0076**	-	**-1.10**	hypothetical protein	Function unknown
**SO_0085**	-	**-1.04**	hypothetical protein	Function unknown
**SO_0139**	ftn	**-1.10**	ferritin	Inorganic ion transport and metabolism
**SO_0147**	-	**-1.35**	hypothetical protein	Function unknown
**SO_0159**	-	**-3.95**	hypothetical protein	Function unknown
**SO_0160**	-	**-1.40**	transporter. putative	Coenzyme transport and metabolism
**SO_0184**	-	**-4.45**	microcystin dependent protein	Function unknown
**SO_0185**	-	**-2.79**	microcystin dependent protein	Function unknown
**SO_0187**	-	**-1.45**	serine protease	Posttranslational modification. protein turnover. chaperones
**SO_0208**	-	**-1.22**	RNA-binding protein	General function prediction only
**SO_0306**	-	**-1.88**	hypothetical protein	Cell wall/membrane/envelope biogenesis
**SO_0313**	potE	**-3.35**	putrescine transporter	Amino acid transport and metabolism
**SO_0346**	-	**-1.04**	GntR family transcriptional regulator	Transcription
**SO_0350**	-	**-1.29**	hypothetical protein	Function unknown
**SO_0365**	-	**-1.45**	major facilitator superfamily permease	Carbohydrate transport and metabolism. Amino acid transport and metabolism. Inorganic ion transport and metabolism. General function prediction only
**SO_0438**	-	**-2.67**	short chain dehydrogenase	Lipid transport and metabolism. Secondary metabolites biosynthesis. transport and catabolism. General function prediction only
**SO_0445**	-	**-2.37**	hflC protein. putative	Posttranslational modification. protein turnover. chaperones
**SO_0449**	-	**-1.31**	hypothetical protein	Function unknown
**SO_0462**	-	**-1.03**	hypothetical protein	Function unknown
**SO_0548**	-	**-1.56**	HU family DNA-binding protein	Replication. recombination and repair
**SO_0571**		**-1.02**	hypothetical protein	
**SO_0670**	-	**-1.37**	hypothetical protein	Function unknown
**SO_0672**	-	**-1.15**	hypothetical protein	Function unknown
**SO_0709**	-	**-3.00**	hypothetical protein	Function unknown
**SO_0710**	-	**-2.41**	hypothetical protein	General function prediction only
**SO_0715**	-	**-1.24**	oxidoreductase. molybdopterin-binding	General function prediction only
**SO_0719**	-	**-1.10**	TonB-dependent receptor. putative	Inorganic ion transport and metabolism
**SO_0764**	-	**-1.18**	hypothetical protein	Function unknown
**SO_0809**	azu	**-1.42**	azurin precursor	Energy production and conversion
**SO_0812**	-	**-1.51**	hypothetical protein	Function unknown
**SO_0813**	-	**-1.30**	hypothetical protein	Amino acid transport and metabolism. Coenzyme transport and metabolism. Cell cycle control. cell division. chromosome partitioning. Cytoskeleton
**SO_0915**	-	**-2.28**	ankyrin domain-containing protein	General function prediction only
**SO_1023**		**-2.21**	NADH dehydrogenase I subunit B	
**SO_1055**	-	**-1.50**	sensory box protein	Signal transduction mechanisms

Table S3: significantly downregulated genes in Δ*uvrY*

ORF	Gene	Regulation[1]	Product	COG description
**SO_1147**		**-1.30**	hypothetical protein	
**SO_1251**	-	**-1.31**	ferredoxin. 4Fe-4S	Energy production and conversion
**SO_1376**	-	**-2.19**	hypothetical protein	Function unknown
**SO_1382**	-	**-2.35**	hypothetical protein	Function unknown
**SO_1397**	-	**-2.37**	cytosine deaminase	Nucleotide transport and metabolism. Translation. ribosomal structure and biogenesis
**SO_1476**	smpA	**-1.13**	small protein A	Translation. ribosomal structure and biogenesis
**SO_1483**	aceB	**-2.00**	malate synthase	Energy production and conversion
**SO_1490**	adhB	**-2.51**	alcohol dehydrogenase II	Energy production and conversion
**SO_1512**	-	**-1.51**	hypothetical protein	Energy production and conversion
**SO_1513**	-	**-2.20**	hypothetical protein	Nucleotide transport and metabolism
**SO_1571**	-	**-1.01**	hypothetical protein	Function unknown
**SO_1651**	-	**-1.07**	Snf2 family protein	Transcription. Replication. recombination and repair
**SO_1661**	-	**-1.40**	LysR family transcriptional regulator	Transcription
**SO_1763**	sat	**-2.09**	streptogramin A acetyl transferase	General function prediction only
**SO_1844**	-	**-2.57**	extracellular nuclease. putative	General function prediction only
**SO_1860**	-	**-5.02**	response regulator	Signal transduction mechanisms. Transcription
**SO_2002**	-	**-2.74**	hypothetical protein	Function unknown
**SO_2073**	hisD	**-2.37**	histidinol dehydrogenase	Amino acid transport and metabolism
**SO_2074**	hisG	**-2.34**	ATP phosphoribosyltransferase	Amino acid transport and metabolism
**SO_2097**	hydC	**-1.11**	quinone-reactive Ni/Fe hydrogenase. cytochrome b subunit	Energy production and conversion
**SO_2102**	-	**-1.33**	hypothetical protein	Function unknown
**SO_2193**	-	**-1.05**	DNA-binding response regulator	Signal transduction mechanisms. Transcription
**SO_2260**	suhB	**-1.05**	extragenic suppressor protein SuhB	Carbohydrate transport and metabolism
**SO_2295**	-	**-1.16**	MATE efflux family protein	Defense mechanisms
**SO_2351**	-	**-1.70**	hypothetical protein	Function unknown
**SO_2565**	-	**-1.75**	intracellular proteinase inhibitor domain-containing protein	Function unknown
**SO_2595**	-	**-1.25**	BNR repeat-containing protein	General function prediction only
**SO_2597**	-	**-1.31**	hypothetical protein	Function unknown
**SO_2725**	-	**-2.58**	LuxR family transcriptional regulator	Transcription
**SO_2856**	-	**-1.55**	CBS domain-containing protein	Signal transduction mechanisms
**SO_2858**	-	**-1.24**	hypothetical protein	Function unknown
**SO_2869**	-	**-1.09**	hypothetical protein	General function prediction only
**SO_2912**	pflB	**-1.55**	formate acetyltransferase	Energy production and conversion
**SO_2913**	pflA	**-2.85**	pyruvate formate lyase-activating enzyme 1	Posttranslational modification. protein turnover. chaperones

Table S3: significantly downregulated genes in Δ*uvrY*

ORF	Gene	Regulation[1]	Product	COG description
**SO_2915**	ackA	**-1.95**	acetate kinase	Energy production and conversion
**SO_2924**	-	**-1.07**	signal peptidase I family protein	Intracellular trafficking. secretion. and vesicular transport
**SO_3019**	trpE	**-1.32**	anthranilate synthase component I	Amino acid transport and metabolism. Coenzyme transport and metabolism
**SO_3020**	trpG	**-1.46**	anthranilate synthase component II	Amino acid transport and metabolism. Coenzyme transport and metabolism
**SO_3043**	-	**-1.60**	hypothetical protein	Amino acid transport and metabolism
**SO_3063**	-	**-1.58**	sodium:alanine symporter family protein	Amino acid transport and metabolism
**SO_3091**	-	**-1.07**	hypothetical protein	General function prediction only
**SO_3102**	-	**-1.10**	AcrA/AcrE family protein	Cell wall/membrane/envelope biogenesis
**SO_3103**	-	**-1.39**	AcrB/AcrD/AcrF family protein	Defense mechanisms
**SO_3104**	-	**-1.13**	hypothetical protein	Inorganic ion transport and metabolism
**SO_3171**	-	**-1.90**	polysaccharide biosynthesis protein	Cell wall/membrane/envelope biogenesis. Carbohydrate transport and metabolism
**SO_3179**	-	**-1.70**	lipopolysaccharide biosynthesis polymerase. putative	Function unknown
**SO_3180**	-	**-1.33**	glycosyl transferase. group 2 family protein	Cell wall/membrane/envelope biogenesis
**SO_3182**	-	**-1.06**	acetyltransferase	Transcription. General function prediction only
**SO_3185**	-	**-1.31**	polysaccharide biosynthesis protein	Cell wall/membrane/envelope biogenesis
**SO_3188**	rfbB	**-1.22**	dTDP-glucose 4.6 dehydratase	Cell wall/membrane/envelope biogenesis
**SO_3190**	-	**-1.25**	polysaccharide biosynthesis protein	Cell wall/membrane/envelope biogenesis
**SO_3444**	-	**-1.29**	hypothetical protein	Amino acid transport and metabolism
**SO_3480**	-	**-2.05**	phosphatase	Signal transduction mechanisms
**SO_3556**	-	**-2.27**	cyclic nucleotide phosphodiesterase. putative	Signal transduction mechanisms
**SO_3645**	-	**-1.03**	hypothetical protein	Function unknown
**SO_3794**	-	**-1.11**	hypothetical protein	Function unknown
**SO_3800**	-	**-1.72**	serine protease	Posttranslational modification. protein turnover. chaperones
**SO_4003**	-	**-1.30**	response regulator	Transcription. Signal transduction mechanisms
**SO_4026**	-	**-1.07**	hypothetical protein	Function unknown
**SO_4043**	-	**-2.91**	TonB domain-containing protein	Cell wall/membrane/envelope biogenesis
**SO_4046**	-	**-1.52**	hypothetical protein	Function unknown
**SO_4118**	-	**-1.69**	malate oxidoreductase. putative	Energy production and conversion
**SO_4179**	-	**-1.09**	glycosyl transferase. group 2 family protein	Cell wall/membrane/envelope biogenesis
**SO_4249**	dfp	**-1.19**	DNA/pantothenate metabolism flavoprotein	Coenzyme transport and metabolism
**SO_4252**	-	**-3.22**	prolyl oligopeptidase family protein	Amino acid transport and metabolism
**SO_4270**	-	**-1.41**	hypothetical protein	Function unknown
**SO_4303**	-	**-1.45**	hypothetical protein	Transcription
**SO_4318**	rtxB	**-1.27**	toxin secretion ATP-binding protein	Defense mechanisms

Table S3: significantly downregulated genes in Δ*uvrY*

ORF	Gene	Regulation[1]	Product	COG description
**SO_4322**	-	**-1.99**	hypothetical protein	Function unknown
**SO_4323**	-	**-2.93**	GGDEF domain-containing protein	Signal transduction mechanisms
**SO_4324**	-	**-1.15**	GGDEF domain-containing protein	Signal transduction mechanisms
**SO_4333**	-	**-1.88**	hypothetical protein	Function unknown
**SO_4334**	-	**-1.90**	hypothetical protein	Defense mechanisms
**SO_4342**	-	**-1.14**	hypothetical protein	Function unknown
**SO_4343**	-	**-1.81**	aminotransferase. class V	Amino acid transport and metabolism
**SO_4345**	ilvD	**-1.60**	dihydroxy-acid dehydratase	Amino acid transport and metabolism. Carbohydrate transport and metabolism
**SO_4346**	ilvM	**-1.94**	acetolactate synthase 2 regulatory subunit	Function unknown
**SO_4418**	-	**-2.31**	trypanothione synthetase domain-containing protein	Function unknown
**SO_4419**	-	**-2.41**	hypothetical protein	Amino acid transport and metabolism
**SO_4420**	-	**-2.82**	M24/M37 family peptidase	Cell wall/membrane/envelope biogenesis
**SO_4547**	-	**-1.15**	hypothetical protein	Replication. recombination and repair
**SO_4564**	-	**-1.18**	TonB2 protein. putative	Cell wall/membrane/envelope biogenesis
**SO_4599**	-	**-1.61**	ribonuclease T2 family protein	Translation. ribosomal structure and biogenesis
**SO_4616**	-	**-1.12**	polysaccharide deacetylase family protein	Carbohydrate transport and metabolism
**SO_4618**	-	**-1.73**	prolyl oligopeptidase family protein	Amino acid transport and metabolism
**SO_4628**	-	**-1.51**	sulfatase	Cell wall/membrane/envelope biogenesis
**SO_4664**		**-2.68**	conserved hypothetical protein	
**SO_4665**	-	**-1.95**	hypothetical protein	Function unknown
**SO_4685**	-	**-1.23**	hypothetical protein	Function unknown
**SO_A0047**	-	**-3.19**	hypothetical protein	Function unknown
**SO_A0048**	-	**-2.05**	prolyl oligopeptidase family protein	Amino acid transport and metabolism
**SO_A0049**	-	**-2.28**	toxin secretion ABC transporter. ATP-binding subunit/permease protein. putative	Defense mechanisms
**SO_A0050**	-	**-2.31**	toxin secretion. membrane fusion protein	Cell wall/membrane/envelope biogenesis
**SO_A0176**	-	**-2.00**	hypothetical protein	Function unknown

[1]log fold change
