# Supplementary material for: Analysis of the BarA/UvrY Two-Component System in Shewanella oneidensis MR-1
Source: PLoS One. 2011 Sep 12;6(9):e23440. doi: 10.1371/journal.pone.0023440 (PMC3171408; doi:10.1371/journal.pone.0023440)
Supplement: Table S4 — Primer used in this study. (PDF) [file pone.0023440.s008.pdf]

Table S4: Primers used in this study

Generation of vectors for in-frame deletions/complementations

BamHI-dSO3457-us-Fw	AGG ATC CAG GGG CTA AAG AAC CTT CGG C
dSO3457-us-Rev	CGC TGA TAG CAC CAG AAC CCA AGA ACG TAA GC
dSO3457-ds-Fw	GTT CTG GTG CTA TCA GCG GAA ATA ACA GAT GAC C
EcoRI-dSO3457-ds-Rev	CGA ATT CGG CGG TAA TTT TGG GAT T
PspOMI-uvrY-us-Fw	TAT CTG GGC CCT GGA AAG CCA TCA GCA ATA ATG GC
uvrY-us-Rev	TTT ATA GCG CCC AGT TCT TAC CAG CTC ATG ATC
uvrY-ds-Fw	AGA ACT GGG CGC TAT AAA ATG TTA GAT GCT GGA CAT T
EcoRI-uvrY-ds-Rev	CGA ATT CAT GGT AAG CAA GGC GTG GAT GC

Generation of vectors for protein production

EcoRI-NOE-uvrY-Fw	CGA ATT CTT GAT ATC GAT ATA TTT AGT TGA CGA TCA TG
XmaI-uvrY-Rev	GAC CCG GGA TTA ATT AAT GCT ATA GAT TAA AAA TGT CCA GC
uvrY_point-mutation_D-N_For	CGT CAT CTT AAT G**A**A CAT GAA TAT GCC
uvrY_point-mutation_D-N_Rev	GGC ATA TTC ATG T**T**C ATT AAG ATG ACG
XhoI-SO3457-HAMP-C-Term-Fw	TCT CGA GGC CGC CTT TAT TAT CGT GTT GAT TG
EcoRI-SO3457-C-Term-Rev	CGA ATT CTT AAA GAA GCC CGT TTT TTT GGT CAT C
barA_point-mutation_H-A_For	CTA ATA TGT CC**G C**CG AGC TGC G
barA_point-mutation_H-A_Rev	CGC AGC TCG **GC**G GAC ATA TTA G

q-RT-PCR

gacA-Q-PCR-077-Fw	GGA TCA AAG TTG TCG GTG AAG CG
gacA-Q-PCR-232-Rev	TCT TGG CAT GGG GTT GAT AAC GC
csrB1-Q-PCR-Fw	CGT CTA TGG AAG GTA TTG AGC AGG
csrB1-Q-PCR-Rev	CGT CCT ACC ATC CCT GCA TCC
csrB2-Q-PCR-Fw	GGA TGA AAG CGA TAC GAA CCA CG
csrB2-Q-PCR-Rev	TGA GCG GTT CCA ACA TCA TCC G
csrB(Sput)-Q-PCR-Fw	ATG CAA CAG GAC TAA GCC ATA GG
csrB1(Sput)-Q-PCR-Rev	TGC ACC ATC CAT GCG TCC G
csrB2(Sput)-Q-PCR-Fw	GCG ATT GAA AGG ATG CTA TTT ACC C
csrB2(sput)Q-PCR-Rev	ATT CGA GCA TCC TGC ATC ATC C
csrB1(Sspec MR-7)-Q-PCR-Fw	GAT GCA GGA AGT ATC GAG TAA AAG G
csrB1(SspecMR-7)-Q-PCR-Rev	TTC CCT GTG CAG TGA AGT CTC C
csrB2(Sspec MR-7)-Q-PCR-Fw	CAG GAT GAA AGC GAT GCG AAC C
csrB2(SspecMR-7)-Q-PCR-Rev	CCT TTT ACC AAG CTT GCT CTT TCC
SO_4706_Malate_syn-qPCR-For	CGA ATC TAA ACA GCT TCA TCG CC
SO_4706_Malate_syn-qPCR-Rev	CCA CAT AGC TGA TCA CCT CTT GG

SO_1483_Malate_syn_A-qPCR-For	GCC AAG CGT AAA GAT AGA CAA GCG
SO_1483_Malate_syn_A-qPCR-Rev	GTT CAA CGG GGC CGG TAA TTT C
SO_1484_Isocitrate_ly_qPCR_For	CTT CGT GGT TCA ATC GTA CCC G
SO_1484_Isocitrate_ly_qPCR_Rev	CGC TTT AGC TTG CTG TAC CGC
SO_2915_Acetat_kinase_qPCR-For	CGT CAA TAA AAT CCT CGC AGG TC
SO_2915_Acetat_kinase_qPCR-Rev	GGA TTA TGC AGT GGC GCT AGG
SO_0184_Microcystin_A_qPCR_For	CGA AGT GCG ATG GCA GCT TG
SO_0184_Microcystin_A_qPCR_Rev	CGA TCG CTA ACA CCC ACA TGC
SO_0185_Microcystin_B_qPCR_For	GCC AAT CAG TCA GTA CGA GGC
SO_0185_Microcystin_B_qPCR_Rev	AAC TCT GCC CGA TAA TTC GAT TGG
csrC-qPCR-Fw	GCG AAT AGC TTT GGA TGC ACT GG
csrC-qPCR-Rev	GCC ATC CTG ACC ACA GTT TCC

Generation of Northern probes

csrB1-Northern-Fw	CAG GAT ACA ATG CTT GTT AGG ACG
csrB1-Northern-Rev	CCT GCT TAT TCG TGC TCC CTG
csrB2-Northern-Fw	GGA TGT TAG CGG TCA GGA TGA C
csrB2-Northern-Rev	CCT GCT TCA TCC CTG TTC AAT CC
